# CO‐Free Aminocarbonylation of Terminal Alkynes Catalyzed by Synergistic Effect From Metal–Organic Frameworks

**DOI:** 10.1002/advs.202405308

**Published:** 2024-09-05

**Authors:** Jian Zhao, Tianze Zhang, Hang Xu, Sheng‐Li Hou, Fang‐Yu Ren, Jie Han, Bin Zhao

**Affiliations:** ^1^ Department of Chemistry Key Laboratory of Advanced Energy Materials Chemistry Renewable Energy Conversion and Storage Center (RECAST) Nankai University Tianjin 300071 P. R. China

**Keywords:** 100% atom economy, CO‐free aminocarbonylation, metal–organic frameworks, mild conditions, synergistic catalysis

## Abstract

Incorporation of CO into substrates to construct high‐value carbonyl compounds is an intensive industrial carbonylation procedure, however, high toxicity and wide explosion limits (12.5–74.0 vol% in air) of CO limit its application in industrial production. The development of a CO‐free catalytic system for carbonylation is one of ideal methods, but full of challenge. Herein, this study reports the CO‐free aminocarbonylation conversion of terminal alkynes synergistically catalyzed by a unique Co(ІІ)/Ag(І) metal–organic framework (MOF), in which the combination of isocyanides and O_2_ is employed as safe and green source of aminocarbonyl. This reaction has broad substrate applicability in terminal alkyne and isocyanides components with 100% atom economy. The bimetal MOF catalyst can be recycled at least five times without substantial loss of catalytic activities. Mechanistic investigations demonstrate that the synergistic effect between Ag(I) and Co(II) sites can efficiently activate terminal alkyne and isocyanides, respectively. Free radical capture experiments, FT‐IR analysis and theoretical explorations further reveal that terminal alkynes and isocyanides can be catalytically transformed into an anionic intermediate through heterolysis pathways. This work provides secure and practical access to carbonylation as well as a new approach to aminocarbonylation of terminal alkynes.

## Graphical abstract

This study proposes CO‐free aminocarbonylation of terminal alkynes catalyzed by a bimetallic Co(II)/Ag(І) metal–organic framework, along with 100% atom economy, safe aminocarbonyl source and mild condition. The excellent catalytic performance can be attributed to synergistic effect of Co(II)/Ag(І) metal–organic framework with modified Ag(І) sites and intrinsic Co(II) sites effectively activates substrates during catalytic reaction.

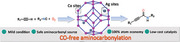

## 1 Introduction

Carbonylation is a fundamental and essential industrial production method due to ubiquitous utilization of carbonyl‐containing compounds in the synthesis of pharmaceuticals,^[^ ^1^ ^]^ natural products,^[^ ^2^, ^3^, ^4^ ^]^ and chemicals.^[^ ^5^ ^]^ On an industrial scale, classical carbonylation processes including oxidative carbonylation, alkoxycarbonylation, double carbonylation, and aminocarbonylation have been extensively utilized.^[^ ^6^, ^7^ ^]^ Nevertheless, the above‐mentioned carbonylation reactions are mainly reliant on palladium‐catalyzed oxidative carbonylation with the mixture of CO and air. A wide range of explosibility limits (12.5–74.0 vol% in air) of CO easily trigger severe fire and explosion accidents.^[^ ^8^ ^]^ Additionally, the affinity of CO with human hemoglobin molecules is 230 times higher than oxygen.^[^ ^9^ ^]^ Inhaling high quantities of CO substantially reduces the delivery of oxygen by hemoglobin, resulting in chemical asphyxia. Over the past decades, CO risk has been a continuing international issue for concern, and may be increasingly aggravated in economically underdeveloped countries.^[^ ^10^ ^]^ Indeed, seeking safe and readily accessible CO precursors to accomplish CO‐free carbonylation is highly desirable and important for the protection of human health and safety. In these explorations, some outstanding progress of CO‐free aminocarbonylation of terminal alkynes has been made, including molybdenum carbonyl complexes^[^ ^11^ ^]^ or chloroform^[^ ^12^ ^]^ as CO surrogates. Nevertheless, metal carbonyl complexes and Pd‐based catalysts have toxicity and produce copious metal waste after catalytic reaction, which limit their widespread use in industry.^[^ ^6^, ^13^, ^14^, ^15^, ^16^, ^17^, ^18^ ^]^ Therefore, developing a low‐cost and eco‐friendly catalyst for CO‐free aminocarbonylation of terminal alkynes is crucial but still challenging.

Metal–organic frameworks (MOFs) are a type of self‐assembly porous materials from metal cations and bridging linkers. Benefited from high tunability, functional diversity and exposed active sites,^[^ ^19^, ^20^, ^21^, ^22^, ^23^, ^24^, ^25^, ^26^, ^27^, ^28^, ^29^, ^30^, ^31^, ^32^, ^33^, ^34^, ^35^, ^36^, ^37^, ^38^, ^39^, ^40^, ^41^, ^42^, ^43^ ^]^ MOFs have attracted intensive interest in various catalytic reactions as heterogenous catalysts, including carbonylation and CO‐free carbonylation.^[^ ^5^, ^41^, ^42^ ^]^ For example, Zhang et al. integrated multimetallic catalytic sites (Co single‐site and Cu‐Pd nanocluster) into MOFs to develop MOF composite photocatalysts for carbonylation reactions without CO.^[^ ^41^ ^]^ However, aminocarbonylation of terminal alkynes, an important branch of carbonylation, still limited to transition metal catalysts with reactants and CO. Compared with traditional transition‐metal catalysts, the interior of MOFs enables highly porous structure and oriented design of catalytically multimetallic sites, which are contribute to the development of CO‐free aminocarbonylation of terminal alkynes. Thus, rational design of introducing appropriate catalytic sites into MOFs is a potential approach to achieve efficient heterogeneous catalysis for CO‐free aminocarbonylative transformations of terminal alkynes.

**Scheme 1:**
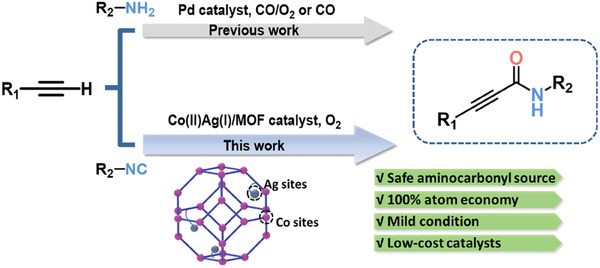
Aminocarbonylation of terminal alkynes: Previous work and our procedure.

In this work, we prepared a histidine‐functionalized MOF and further modified with Ag(І) sites to construct a bimetallic Co(II)/Ag(І) catalyst (L‐ZIF‐67‐Ag‐0.3). The L‐ZIF‐67‐Ag‐0.3 catalytic system enables CO‐free aminocarbonylation of terminal alkynes with 100% atomic economy by using O_2_ and isocyanide as sources of aminocarbonyl,^[^ ^44^ ^]^ which are less toxic and safer than CO (**Scheme** **1**). The catalytic results show that the unique structure of L‐ZIF‐67‐Ag‐X exhibits excellent catalytic performance and substrate universality. L‐ZIF‐67‐Ag‐X can be easily recycled and reused at least five times without producing any waste after catalytic reaction. Meanwhile, L‐ZIF‐67‐Ag‐0.3 exhibited excellent catalytic properties for CO‐free aminocarbonylation of terminal alkynes and the amount of Ag is only 3.69 mol% during the reaction. It is effective to reduce catalyst costs by replacing Pd with Ag, since Pd is currently ≈100 times more expensive than Ag.^[^ ^45^ ^]^ NMR spectroscopy reveals that synergistic effect of L‐ZIF‐67‐Ag‐X with modified Ag(І) sites and intrinsic Co(II) sites effectively activates substrates during catalytic reaction. A feasible heterolysis pathway of the intermediate product was verified by free radical capture experiments and computational studies. Most predominantly, it is the first example of CO‐free aminocarbonylation of terminal alkynes with 100% atom economy. This study opened a new avenue for CO‐free aminocarbonylation of terminal alkynes.

## 2 Results and Discussion

**Figure 1:**
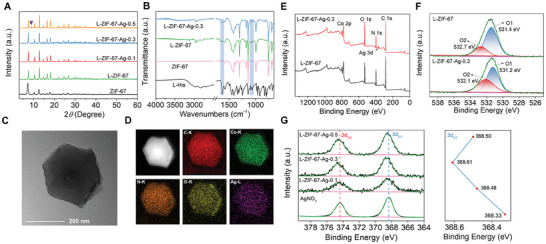
A) XRD patterns of the synthesized ZIF‐67, L‐ZIF‐67, L‐ZIF‐67‐Ag‐0.1, L‐ZIF‐67‐Ag‐0.3, and L‐ZIF‐67‐Ag‐0.5. B) FT‐IR spectra of L‐His, ZIF‐67, L‐ZIF‐67, L‐ZIF‐67‐Ag‐0.3. C) TEM of L‐ZIF‐67‐Ag‐0.3. D) Dark field STEM and elemental mapping images of L‐ZIF‐67‐Ag‐0.3. E) The XPS spectra of L‐ZIF‐67 and L‐ZIF‐67‐Ag‐0.3. F) XPS analysis of O1s regions for L‐ZIF‐67 and L‐ZIF‐67‐Ag‐0.3. G) XPS analysis of Ag 3d regions for AgNO_3_, L‐ZIF‐67‐Ag‐0.1, L‐ZIF‐67‐Ag‐0.3 and L‐ZIF‐67‐Ag‐0.5.

According to the XRD patterns, characteristic diffraction peaks at ≈7.4°, 10.4°, and 12.8° were observed with pristine ZIF‐67, L‐ZIF‐67, L‐ZIF‐67‐Ag‐0.1, L‐ZIF‐67‐Ag‐0.3, and L‐ZIF‐67‐Ag‐0.5 samples (**Figure** **1**A). These samples showed resemble diffraction peaks with ZIF‐67, indicating the obtained samples have similar crystal structures and Ag(І) only coordinated with exposed carboxyl and amino groups without being incorporated into L‐ZIF‐67 framework.^[^ ^46^ ^]^ Nevertheless, two diffraction peaks at 8.9° and 12.0° were observed with the L‐ZIF‐67‐Ag‐0.5 sample and its intensity is very low (labeled by triangle in Figure 1A). We presumed that as the increase of Ag loadings, the available coordination sites for carboxyl and amino groups gradually decreased. Excessive Ag(І) caused the cleavage of the Co‐N bond and generated silver imidazolate oligomers.^[^ ^47^, ^48^ ^]^ FT‐IR spectra were employed to examine as‐synthesized samples structure (Figure 1B; Figure S1, Supporting Information). The absorption peaks of L‐ZIF‐67 and L‐ZIF‐67‐Ag‐X showed the C═O stretching at 1629 cm^−1^ in the carboxyl group and amine group at 1059 cm^−1^, which is consistent with the functional groups of L‐histidine. In contrast, these two absorption peaks were unobservable for ZIF‐67. The FT‐IR results verified that L‐His was incorporated into L‐ZIF‐67. The transmission electron microscopy (TEM) images of L‐ZIF‐67 showed regular dodecahedron morphology, with an average diameter of ≈300 nm (Figure S2, Supporting Information). Furthermore, the characteristic elements of C, N, O, and Co can be observed from the scanning TEM (STEM) elemental mapping characterization (Figure S3, Supporting Information). After being coordinated with Ag(І), L‐ZIF‐67‐Ag‐0.3 still remained the well‐defined crystal structure, revealing that the introduction of Ag(І) had no influence on the framework integrity (Figure S4, Supporting Information). The elemental mapping images of L‐ZIF‐67‐Ag‐0.3 further confirm the existence of Ag(І) and uniformly distributed in L‐ZIF‐67 without aggregation (Figure 1C,D). Similarly, L‐ZIF‐67‐Ag‐0.1 and L‐ZIF‐67‐Ag‐0.5 samples showed the dodecahedron crystal and corresponding element composition (Figures S5 and S6, Supporting Information). Moreover, no deposition of Ag nanoparticles was observed in TEM images of the L‐ZIF‐67‐Ag‐X samples (Figure 1C,D; Figures S5 and S6, Supporting Information).

To further characterize L‐ZIF‐67 and coordination states of Ag species in the L‐ZIF‐67‐Ag‐X samples, all of the samples were conducted by X‐ray photoelectron spectroscopy (XPS) measurements. Carboxylic group of L‐ZIF‐67 was identified from XPS analysis and emerged at 288.2 eV in the C1s spectrum (Figure S7, Supporting Information).^[^ ^49^ ^]^ The related elements of L‐ZIF‐67 and L‐ZIF‐67‐Ag‐X fit well with the photoelectron peaks (Figure 1E; Figure S8, Supporting Information). The O1s XPS spectra of O1 and O2 with 531.4 eV and 532.7 eV are assigned to C═O and C─O bonds, respectively.^[^ ^50^, ^51^ ^]^ The binding energies of O1s show significant variation in comparison to L‐ZIF‐67 and L‐ZIF‐67‐Ag‐X, which indicated Ag(I) were successfully coordinated with the carboxyl group (Figure 1F). N1s region of XPS analysis further verified the C‐NH_2_ peak shift is resulted from the coordination disturbance between Ag(І) and amino group (Figure S9, Supporting Information). Notably, the Ag3d region of L‐ZIF‐67‐Ag‐X shows two typical peaks ≈368 and 375 eV, which can be attributed to Ag(I) 3d_5/2_ and Ag(I) 3d_3/2_ electronic configurations, respectively (Figure 1G). With the increase of the Ag content, the high‐resolution Ag3d_5/2_ and Ag3d_3/2_ spectrum shifts toward higher binding energy (L‐ZIF‐67‐Ag‐0.3 > L‐ZIF‐67‐Ag‐0.1 > AgNO_3_), exhibiting the modified local electronic structure of Ag(I). Accordingly, the results demonstrate that Ag(I) was successfully introduced into L‐ZIF‐67, being coordinated with amino acid groups. However, the Ag3d_5/2_ XPS characteristic peaks of L‐ZIF‐67‐Ag‐0.5 shifted to 368.50 eV, lower than L‐ZIF‐67‐Ag‐0.3, which could be ascribed to the formation of silver imidazolate oligomers. To gain more insights into the local coordination environment of the Ag ion of MOF catalysts,^[^ ^45^ ^]^ Raman spectroscopy experiments were conducted on corresponding MOF catalysts including ZIF‐67, L‐ZIF‐67 and L‐ZIF‐6‐Ag‐0.3 (Figure S10, Supporting Information). Compared with ZIF‐67, the appearance of a new peak in the spectrum of L‐ZIF‐6 at 746 cm^−1^, which be assigned to O─C═O bending vibration. The peak intensity at 746 cm^−1^ of L‐ZIF‐6‐Ag‐0.3 decreases slightly, indicating that the O atom of the carboxyl group is involved in the bond formation with Ag(I). For L‐ZIF‐67 and L‐ZIF‐6‐Ag‐0.3, a broad band at 1205 cm^−1^ appears owing to the N–H vibration, as well as the peak ≈1365 cm^−1^ is related to C═O stretching vibration.^[^ ^52^ ^]^ With the incorporation of Ag(I), the peaks of C═O stretching vibration of L‐ZIF‐6‐Ag‐0.3 shift from 1365.13 to 1366.58 cm^−1^, which could be ascribed to the formation of Ag─O bonds. The above results demonstrated Ag(I) ions were successfully coordinated with amino acid groups.

The porosity of L‐ZIF‐67 and L‐ZIF‐67‐Ag‐X was further investigated by N_2_ adsorption‐desorption isotherms measurement at 77 K (Figure S11, Supporting Information). Typical type I adsorption isotherms among activated L‐ZIF‐67 and L‐ZIF‐67‐Ag‐X samples were observed according to the IUPAC classification, confirming the microporous nature of the synthesized materials. The N_2_ isotherms show that the BET surface areas of L‐ZIF‐67, L‐ZIF‐67‐Ag‐0.1, L‐ZIF‐67‐Ag‐0.3 and L‐ZIF‐67‐Ag‐0.5 are 1448.8, 1243.1, 1129.2, and 1075.8 m^2^ g^−1^, respectively. Obviously, the introduction of Ag(I) reduced the surface areas and the pore sizes of the resulting L‐ZIF‐67‐Ag‐X. Large BET surface area and high porosity of L‐ZIF‐67‐Ag‐X samples are conducive to the enhanced enrichment of substrates in catalytic reaction. To explore the thermal stability of L‐ZIF‐67 and L‐ZIF‐67‐Ag‐X, the thermogravimetric analysis (TGA) were performed under N_2_ atmosphere. The thermodynamic curve of L‐ZIF‐67 and L‐ZIF‐67‐Ag‐X were found to be identical, with 5% weight loss before 200 °C (Figure S12, Supporting Information), suggesting the post‐synthesis modification method had no effect on the thermal stability of the L‐ZIF‐67‐Ag‐X samples.

By choosing ethynylbenzene **1a** and *tert*‐butyl isocyanide **2a** as the model substrates, the catalytic reactivity of all L‐ZIF‐67‐Ag‐X catalysts and related homogeneous catalysts were studied (**Table** **1**). The substrates did not react in the absence of catalysts (Table 1, entry 1). Subsequently, several control experiments exhibited that the reaction cannot proceed with catalyst of Co(NO_3_)_2_·6H_2_O, AgNO_3_, ZIF‐67 or L‐ZIF‐67 (Table 1, entries 2–5). The targeted product N‐(*tert*‐butyl)−3‐phenylpropiolamide **3a** was obtained from L‐ZIF‐67‐Ag‐X and Co(NO_3_)_2_·6H_2_O+AgNO_3_, indicating that Co(II) and Ag(I) exist positive synergistic effect. Among them, the catalyst L‐ZIF‐67‐Ag‐0.3 was found to be superior to the others, which affording the desired products **3a** in 93% yield (Table 1, entries 6–9), and the yield of corresponding homogeneous catalyst Co(NO_3_)_2_·6H_2_O+AgNO_3_ was only 53%. In addition, the different amounts of Co(NO_3_)_2_·6H_2_O+AgNO_3_ corresponding to the loading of L‐ZIF‐67‐Ag‐0.1 and L‐ZIF‐67‐Ag‐0.5 were attempted under optimized conditions (Table 1, entry 6), providing product yield in 23% and 61%, respectively. After determining L‐ZIF‐67‐Ag‐0.3 as the optimal catalyst, we further evaluated the effects of the tested solvents. CH_3_CN exhibited the best yield among the selected solvents (Table 1, entries 9–13). Notably, the target product was not obtained in the absence of oxygen, indicating that O_2_ as an oxidant played a crucial role in the reaction (Table 1, entry 14). The catalytic activity of L‐ZIF‐67‐Ag‐0.3 was performed without PPh_3_ and provided product yield in 74% (Table 1, entry 15). In the presence of PPh_3_, the target product **3a** was provided in 93% yield (Table 1, entry 9), indicating PPh_3_ can improve catalytic efficiency. The introduction of PPh_3_ might result in electron‐rich Ag sites, which contribute to the activation of the triple bond of alkynes and further improve the catalytic performance.^[^ ^53^] Therefore, the optimal reaction conditions were determined to be ethynylbenzene **1a** (0.5 mmol), tert‐butyl isocyanide **2a** (0.6 mmol), L‐ZIF‐67‐Ag‐0.3 (50 mg), PPh_3_ (20 mol%), 1 atm. oxygen atmosphere in CH_3_CN (2 mL) at 60 °C for 12 h.

**Table 1:** Optimization of the reaction conditions.^a)^

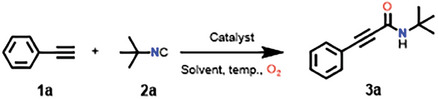			
Entry	Catalyst	Solvent	Yield[%]^b)^
1	–	CH_3_CN	N.R.
2	Co(NO_3_)_2_·6H_2_O	CH_3_CN	N.R.
3	AgNO_3_	CH_3_CN	N.R.
4	ZIF‐67	CH_3_CN	N.R.
5	L‐ZIF‐67	CH_3_CN	N.R.
6	Co(NO_3_)_2_·6H_2_O+AgNO_3_	CH_3_CN	53 (23^c)^, 61^d)^
7	L‐ZIF‐67‐Ag‐0.1	CH_3_CN	56
8	L‐ZIF‐67‐Ag‐0.5	CH_3_CN	80
9	L‐ZIF‐67‐Ag‐0.3	CH_3_CN	93
10	L‐ZIF‐67‐Ag‐0.3	DMSO	trace
11	L‐ZIF‐67‐Ag‐0.3	Dioxane	67
12	L‐ZIF‐67‐Ag‐0.3	DMF	50
13	L‐ZIF‐67‐Ag‐0.3	EtOH	21
14^e)^	L‐ZIF‐67‐Ag‐0.3	CH_3_CN	N.D.
15^f)^	L‐ZIF‐67‐Ag‐0.3	CH_3_CN	74

^a)^ Reaction conditions: **1a** (0.5 mmol), **2a** (0.6 mmol), L‐ZIF‐67‐Ag‐X (50 mg, the amounts of Co and Ag of other catalysts being applied to the reaction correspond to L‐ZIF‐67‐Ag‐0.3), PPh_3_ (20 mol%), solvent (3 mL), 1 atm. oxygen atmosphere, 60 °C and 12 h.

^b)^ All yields are isolated.

^c)^ The amounts of Co and Ag of Co(NO_3_)_2_·6H_2_O+AgNO_3_ used for the reaction correspond to L‐ZIF‐67‐Ag‐0.1.

^d)^ The amounts of Co and Ag of Co(NO_3_)_2_·6H_2_O+AgNO_3_ used for the reaction correspond to L‐ZIF‐67‐Ag‐0.5.

^e)^ 1 atm. argon atmosphere.

^f)^ Without PPh_3_.

With the optimal reaction conditions in hand, we next explored the substrate scope of alkyne derivatives (**Table** **2**). A broad range of alkynes proceeded smoothly to afford the desired 2‐ynamides derivatives in moderate to excellent yields. The ethynylbenzene substrates with electron‐donating groups resulted in targeted 2‐ynamides derivatives **3b** and **3c** in over 85% yield. Additionally, the reactions of substrates with electron‐withdrawing groups obtain the corresponding products **3d**–**3f** in 87%, 81%, and 75% yields, respectively. Cycloalkanes and heterocyclic alkynes are also performed for this reaction, and the yield of desired products **3g** and **3h** are 72% and 63%, respectively. In contrast, under the identical conditions, alkyl‐substituted terminal alkynes of corresponding products **3i** were obtained, which provided moderate yield in 55%. It is supposed that the weak nucleophilicity of alkyl‐substituted terminal alkynes impaired the C1 carbon reactivity. Catalytic experiments show that the reaction has good substrate universality for various alkyne derivatives.

**Table 2:** Substrate scope.^a)^

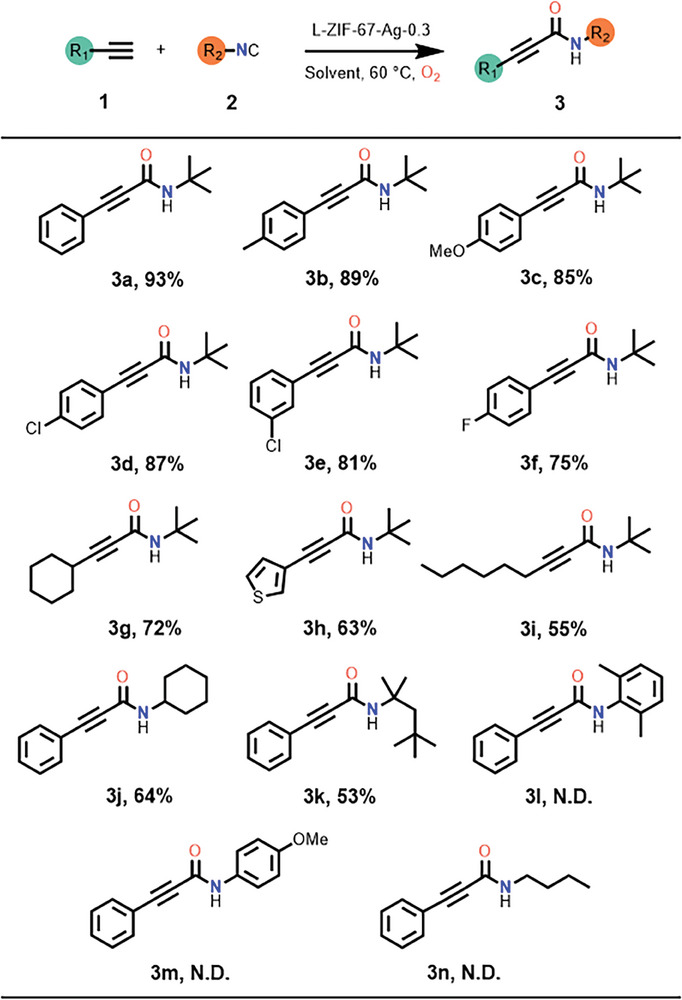

^a)^ Reaction conditions: **1** (0.5 mmol), **2** (0.6 mmol), L‐ZIF‐67‐Ag‐0.3 (50 mg, 3.69 mol% Ag), PPh_3_ (20 mol%), CH_3_CN (3 mL), 1 atm. oxygen atmosphere, 60 °C and 12 h. All yields are isolated.

The substrates of isocyanide derivatives were also investigated under the optimal reaction conditions. Under the standard conditions, the reactions of cyclohexyl isocyanide and *tert*‐octyl isocyanide with ethynylbenzene proceeded smoothly to afford **3j** and **3k** in 64% and 53% yields, respectively. Out of our expectation, the desired products of aromatic isocyanides such as N‐(2,6‐dimethylphenyl)−3‐phenylpropiolamide **3l** and N‐(4‐methoxyphenyl)−3‐phenylpropiolamide **3m** were not detected, possibly because of the increased oxidation potential of the intermediates in the reaction.^[^ ^54^ ^]^ Similarly, the corresponding product **3n** was not obtained when using alkyl‐substituted isocyanide as substrate. These results reveal that the catalyst have catalytic activity for cyclohexyl and *tert*‐octyl isocyanides, but not for aromatic and alkyl isocyanides.

The chemical stability of L‐ZIF‐67‐Ag‐0.3 was evaluated by analysing PXRD in different treatments (Figure S13, Supporting Information). Placing the samples in open air for 7 days, the PXRD pattern showed that no influence could be detected for the L‐ZIF‐67‐Ag‐0.3. Thereafter the L‐ZIF‐67‐Ag‐0.3 samples were tested by incubating in 1 m NaOH aqueous solution (24 h), refluxing acetonitrile (3 days) and boiling water (12 h), as well as in common solvents (DMF, toluene and ethanol) for 7 days. The crystal structures of treated samples are well retained under all the conditions tested. The structure of L‐ZIF‐67‐Ag‐0.3 was decomposed after soaking in HCl solution (1 m) for 24 h. Subsequently, the catalyst L‐ZIF‐67‐Ag‐0.3 was separated via centrifugation from the reaction mixture after the reaction was finished, and its recyclability was investigated (Figure S14, Supporting Information). It can be observed that L‐ZIF‐67‐Ag‐0.3 can be reused at least five times without significant deterioration of the products yield (more than 82%). Both PXRD results revealed that the crystalline structure of L‐ZIF‐67‐Ag‐0.3 exhibited negligible changes after reaction (Figure S15, Supporting Information). The heterogeneous nature of L‐ZIF‐67‐Ag‐0.3 was further evidenced by leaching experiment. The product yield was not significantly increased after removal of the L‐ZIF‐67‐Ag‐0.3 at 3 h (Figure S16, Supporting Information). Quantitative analysis of ICP‐OES showed that the presence of 0.07 ppm Co and 0.4 ppm Ag in the supernatant mixture. The supernatant mixture was further analyzed by (atomic absorption spectroscopy) AAS experiment. Experimental results showed that the presence of 0.07 ppm Co and 0.47 ppm Ag in the supernatant mixture. The above results indicated that no obvious cobalt and silver leaching happened during the reaction. In addition, there was no significant difference in the XPS spectra of Co 2p, Ag 3d, N 1s and O1s regions, which indicated the L‐ZIF‐67‐Ag‐0.3 catalyst was well‐retained before and after catalytic reaction (Figure S17, Supporting Information). All these results suggest that L‐ZIF‐67‐Ag‐0.3 has good catalytic stability.

In order to understand the nature of defect in MOF catalysts, EPR of MOF catalysts were investigated including L‐ZIF‐67, L‐ZIF‐67‐Ag‐0.3 and recycled L‐ZIF‐67‐Ag‐0.3 (Figure S18, Supporting Information). The weak EPR signals of L‐ZIF‐67 and L‐ZIF‐67‐Ag‐0.3 were observed, indicating that some intrinsic defects exist in the framework structure. The enhanced EPR signals of recycled L‐ZIF‐67‐Ag‐0.3 revealed that more defective sites were formed after catalytic reaction.^[^ ^55^ ^]^ Furthermore, TGA of recycled L‐ZIF‐67‐Ag‐0.3 was employed under N_2_ atmosphere (Figure S19, Supporting Information). The thermodynamic curve of L‐ZIF‐67‐Ag‐0.3 and recycled L‐ZIF‐67‐Ag‐0.3 showed a weight loss difference of 3.8% at 800 °C, which could be attributed to the formation of linker vacancy during the catalytic reaction.^[^ ^56^ ^]^ To compare the formation rates of products between the catalytic system presented in this study and conventional systems, we calculated the TOF values and listed them in the revision (Table S1, Supporting Information). The TOF of L‐ZIF‐67‐Ag‐0.3 can reach 2.1 h^−1^ among the reported catalysts for aminocarbonylation of terminal alkynes. The reported catalysts including Mo(CO)_6_, Fe_3_(CO)_12_, Pd/C and PdCl_2_ showed lower TOF than L‐ZIF‐67‐Ag‐0.3, indicating that moderate formation rates of products in L‐ZIF‐67‐Ag‐0.3 catalytic system. Other reported catalysts like PdCl_2_(PPh_3_)_2_, Pd(OAc)_2_, Pd_2_(dba)_3_ and Ir/NiCl_2_ exhibited a good value, but noble metal catalysts and high toxic CO were needed during the reaction.

**Figure 2:**
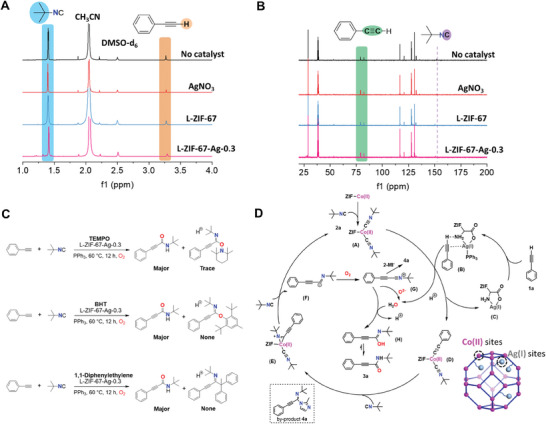
Mechanistic investigation. A) ^1^H NMR and B) ^13^C HMR spectra changes on attempting activation of terminal alkynes by no catalyst, AgNO_3_, L‐ZIF‐67 and L‐ZIF‐67‐Ag‐0.3 systems at 1 atm., oxygen atmosphere (in DMSO‐d_6_). C) Radical capture by TEMPO, BHT and 1,1‐Diphenylethylene under the optimal reaction conditions. D) Mechanistic proposal of the L‐ZIF‐67‐Ag‐0.3 system.

To explore the catalytic mechanism for this reaction, the interactions among substrates and different catalysts were detected by ^1^H NMR and ^13^C NMR spectroscopy under oxygen atmosphere. The ^1^H signal peaks of C≡C─H (*d* = 3.27 ppm) in AgNO_3_ and L‐ZIF‐67‐Ag‐0.3 systems become dwarf in comparison to no catalyst and L‐ZIF‐67 systems, indicating that the alkynyl hydrogen can be activated by AgNO_3_ and L‐ZIF‐67‐Ag‐0.3 systems (**Figure** **2**A). It is worth noting that ^13^C signal peaks of ‐NC in L‐ZIF‐67 and L‐ZIF‐67‐Ag‐0.3 systems shift toward lower position than the other two systems, which may be related to the interactions between ‐NC and Co(II) sites (Figure 2B). Notably, the substrate activation phenomenon was also observed under argon atmosphere (Figures S20 and S21, Supporting Information). Therefore, L‐ZIF‐67‐Ag‐0.3 catalyst achieves this synergistic process of activated substrates without oxygen through Co(II) and Ag(I) sites.

The desired products **3a** were obtained when the addition of 2.5 *equiv* of the radical scavenger 2,2,6,6‐tetramethylpiperidinooxy (TEMPO), 2,6‐di‐tert‐butyl‐4‐methylphenol (BHT) or 1,1‐diphenylethylene under the optimal conditions (Figure 2C). Catalytic results indicated that the reaction proceeded smoothly and the main reaction pathway may not undergo the free radical‐mediated reaction. According to the above results and related literature reports,^[^ ^54^, ^57^, ^58^, ^59^, ^60^ ^]^ a possible reaction mechanism for this reaction is proposed using **1a** and **2a** as model substrates (Figure 2D). First, the metal centers Co(II) in L‐ZIF‐67 interact with **2a** to generate complex **A**. Meanwhile, Ag(I) sites in situ react with PPh_3_, which activate **1a** to afford complex **B**. The bimetallic synergism Co(II) and Ag(I) sites result in the intermediate **D** and a hydrogen proton. The intermediate **D** and **2a** interacted to form complex **E**. **F** was generated from complex **E** through a heterolytic cleavage pathway. Oxygenation of **F** leads to **G** and oxygen anion. Afterward, the formed O^2−^ can extract hydrogen protons from complex **B** to generate H_2_O, and the intermediate **H** was formed by nucleophilic attack of H_2_O. The product **3a** is obtained by tautomerization.

**Figure 3:**
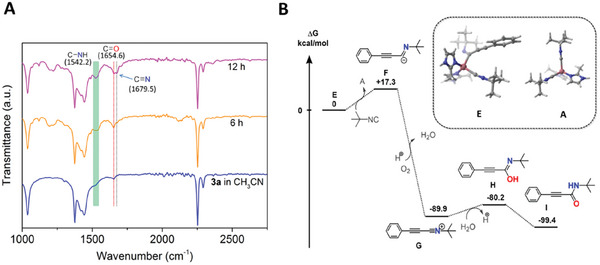
FT‐IR spectra and DFT calculation A) The continuous monitoring of reaction process at different times with FT‐IR spectra. B) Gibbs free energy changes for the steps on the Co centers via the heterolytic cleavage pathway as calculated by DFT.

To further understand the proposed reaction mechanism, we conducted the continuous monitoring of FT‐IR analysis at different time points under the optimal conditions (the amount of substrate was expanded three times to ensure that intermediates and target products were not covered by solvent peaks). The new band at 1654.6 cm^−1^ can be obviously observed at 6 and 12 h, which can be assigned to the C═O stretching vibration (**Figure** **3**A). Additionally, a broad band at 1542.2 cm^−1^ is appeared owing to the C─N stretching vibration and the N─H deformation vibration. These two bands correspond to product **3a** in acetonitrile, indicating that the target products were produced during the reaction process. Meanwhile, the amide bands are gradually strengthened with time, proving that the product gradually increased (Figure S22, Supporting Information). Interestingly, a new band appeared at 1679.5 cm^−1^ at 1 h and it strengthened greatly as the reaction time prolonged, which could be assigned to the C═N stretching vibration (Figure 3A; Figures S22 and S23, Supporting Information). We further evaluated simulated FT‐IR spectra of immediate **F** and found that the band of C═N stretching vibration was observed at 1638.0 cm^−1^ (Figure S24, Supporting Information), which can presumably be ascribed to the formation of immediate **F** during the reaction procedure. Additionally, the catalytic reaction was completed under optimal reaction and the MOF catalyst was separated via filtration. The residual mixture was further purified by flash column chromatography to afford major by‐product **4a** in 4% yield. Owing to the formation of defective sites during catalytic reaction, missing linkers of negative 2‐methylimidazole (2‐MI^−^) nucleophilic attack the intermediate **G**, providing the by‐product **4a**. ^1^H NMR, ^13^C NMR spectra and mass spectrometry of compound **4a** were provided in Figures S25–S27 (Supporting Information). The above results indicated that the formation of intermediate **G** during the reaction.

Based on the above results as well as a unique heterolysis pathway toward this reaction, the relative free energy of stepwise pathway (Path **E**‐**3a**), which is proposed in reaction mechanism, is further evaluated to gain insight. We thus carried out DFT calculations for all of these pathways (Path **E**‐**3a**) in CH_3_CN. The optimized configurations of **A**, **E**‐**H** and **3a** were provided in Figure S28 (Supporting Information). We discovered a mild process of the initial step (ΔG = 17.3 kcal mol^−1^; at 333.15 K), implying that the intermediate **F** can be easily obtained from **E** through the heterolytic cleavage pathway (Figure 3A,B). Notably, the formation of **G** from **F** during oxygenation process was a highly exothermic step by 107.2 kcal mol^−1^, which demonstrated that **F** obtained via the heterolysis cleavage pathway is more likely to form the intermediate **G**. Following that, **G** was nucleophilically extracted from H_2_O to provide the intermediate **H**, which is then tautomerized to form the target product **3a**. The ΔG values of nucleophilic attack and tautomerization exhibited 9.7 kcal mol^−1^ and −19.2 kcal mol^−1^, respectively, indicating that the nucleophilic reaction and tautomerization were both energetically feasible. Therefore, the computed energy profiles confirm that all the computational results discussed above could be accessible for the plausible transformation pathway (Path **E**‐**3a**).

## 3 Conclusion

In summary, we have constructed the stable catalyst L‐ZIF‐67‐Ag‐0.3 which can synergistically catalyze aminocarbonylation of terminal alkynes under mild condition without CO. The catalytic system has a wide range of substrate scope and functional group tolerance, and can be recycled five times without obvious decrease of yields. NMR monitoring further confirmed that Co(II)/Ag(I) sites of L‐ZIF‐67‐Ag‐0.3 catalysts play an essential role in activating substrate to achieve high catalytic efficiency. Free radical capture experiments proved that the primary reaction pathway may undergo a unique anionic intermediate via heterolysis pathways. FT‐IR analysis and computational results established that the heterolysis pathway of the intermediate product is potentially feasible. To our knowledge, this is the first report on CO‐free aminocarbonylation of terminal alkynes with 100% atom economy. This work highlights the feasibility of MOFs to synthesize 2‐ynamides via CO‐free method and broadens synthetic approach to aminocarbonylation of terminal alkynes.

## 4 Experimental Section

Methods and any associated references are available in the Supporting Information.

## Conflict of Interest

The authors declare no conflict of interest.

## Author Contributions

J.Z. and T.Z. contributed equally to this work. J.Z. and T.Z. performed the experiments, analyzed the data, and participated in experiment design. J.Z., T.Z., H.X., and S.H. prepared the manuscript. F.R. provided a discussion on mechanism analysis. J.H. and B.Z. conceived and designed the experiments, supervised the work, and confirmed the manuscript.

## Supporting information

Supporting Information

## Data Availability

The data that support the findings of this study are available in the supplementary material of this article.
