# Supplementary material for: CO‐Free Aminocarbonylation of Terminal Alkynes Catalyzed by Synergistic Effect From Metal–Organic Frameworks
Source: Adv Sci (Weinh). 2024 Sep 5;11(41):2405308. doi: 10.1002/advs.202405308 (PMC11538656; doi:10.1002/advs.202405308)
Supplement: Supplementary file 1 — Supporting Information [file ADVS-11-2405308-s001.pdf]

ADVANCED SCIENCE
Open Access

Supporting Information

for *Adv. Sci.*, DOI 10.1002/advs.202405308

CO-Free Aminocarbonylation of Terminal Alkynes Catalyzed by Synergistic Effect From Metal–Organic Frameworks

Jian Zhao, Tianze Zhang, Hang Xu, Sheng-Li Hou, Fang-Yu Ren, Jie Han and Bin Zhao**

Supporting Information

CO-Free Aminocarbonylation of Terminal Alkynes Catalyzed by Synergistic Effect from Metal-Organic Frameworks

Jian Zhao, Tianze Zhang, Hang Xu, Sheng-Li Hou, Fang-Yu Ren, Jie Han, and Bin Zhao**
Department of Chemistry, Key Laboratory of Advanced Energy Materials Chemistry, Renewable Energy Conversion and Storage Center (RECAST), Nankai University, Tianjin 300071, P. R. China
*Correspondence: hanjie@nankai.edu.cn, zhaobin@nankai.edu.cn

This PDF file includes:

Supplementary Text
Figure S1 to S27
Spectroscopic Data of Compounds
Scanned ^{1}H NMR, and ^{13}C NMR spectra of compounds
References (1 to 23)

Materials and Methods

General information.

All reagents including cobalt nitrate hexahydrate ($Co(NO_3)_2·6H_2O$), silver nitrate ($AgNO_3$), 2-methylimidazole (Hmim), L-histidine (L-His), trimethylamine (TEA), 2,2,6,6-tetramethyl-1-piperidinyloxy (TEMPO), butylated hydroxytoluene (BHT), 1,1-diphenylethylene, methanol, deionized water, ethanol, CH_3CN, PPh_3, DMSO, Dioxane, DMF, petroleum ether (PE), ethyl acetate (EA), $CDCl_3$, DMSO-d_6, terminal alkynes derivatives, isocyanide derivatives, and O_2 (99.9%) were purchased commercially and used as provided without further purification.

Transmission electron microscopy (TEM) measurements were obtained using a JEOL JEM-2100F and FEI Tecnai G2F30. Scanning transmission electron microscope (STEM) elemental

mapping was performed using the same electron microscope equipped with an energy dispersive spectrometry (EDS). XRD patterns were conducted with Rigaku SmartLab X-Ray Diffractometer (9 kW). The oxidation state of the samples was obtained using XPS with an X-ray source of monochromated Al Kα using a theta probe AR-XPS System (Kratos Analytical Ltd., U.K.). Elemental quantity was analyzed by inductively coupled plasma optical emission spectrometry (ICP-OES) using Agilent 725. The Fourier Transform Infrared Spectra (FTIR) were carried out by Nicolet IS10 spectrometer. Gas (N_2) sorption analysis of the samples was conducted at 77 K using V-Sorb 2800TP. Thermogravimetric analysis were collected by a Netzsch TG 209 TG-DTA analyzer. Element analysis was conducted by Elementar Vario EL. Electron paramagnetic resonance (EPR) signals were recorded at room temperature (298 K) with a Bruker ESR A300 spectrometer. Raman spectrum was recorded on a Horiba Evolution (Horiba, France) Raman microscope. AAS experiment was employed using Agilent 240 AA. High resolution mass spectra were obtained on a Varian QFT-ESI mass spectrometer. The products were purified by column chromatography over silica gel, visualized by irradiation with UV light. ^{1}H-NMR and ^{13}C-NMR were recorded on a 400 MHz Bruker 400 spectrometer in $CDCl_3$ or DMSO-d_6, and TMS was used as internal standard.

Synthesis of ZIF-67. ZIF-67 was prepared according to a recent report,[1] 4 mmol $Co(NO_3)_2 \cdot 6H_2O$ dissolved in 100 mL methanol was quickly added to a solution containing 16 mmol 2-methylimidazole in 100 mL methanol, then the solution was aged at room temperature for 24 h. The resulting material was collected by centrifugation, washed with methanol three times, and vacuum dried overnight.

Synthesis of L-ZIF-67. Firstly, 4 mmol L-His and 20 mmol 2-methylimidazole were dissolved in 43 mL H_2O, followed by 2.6 mL TEA was carried out under magnetic stirring. Then, 6 mmol $Co(NO_3)_2 \cdot 6H_2O$ dissolved in 11 mL H_2O was introduced into the above solution, and the mixture was stirred for 30 min at room temperature. The resulting product was collected by centrifugation, washed with H_2O and methanol for 3 times and vacuum dried at 60 °C overnight.

Synthesis of L-ZIF-67-Ag-X. 0.5 g L-ZIF-67 was dispersed into 20 mL ethanol solution and ultrasonic for 10 min. Various amounts of silver nitrate (17, 51 and 85 mg) were dissolved in 30 mL of ethanol and was added into the above solution stirred for 1 h at room temperature. The product was collected by centrifugation at 10000 rpm for 5 min and repeatedly washed with ethanol 3 times. The collected purple powder was vacuum-dried at 60 °C overnight. The obtained samples were named as L-ZIF-67-Ag-X, where X = 0.1, 0.3 and 0.5, corresponding to the molar amount of silver nitrate in the mixture solution. The contents of Co and Ag of L-ZIF-67-Ag-X were analyzed by ICP-OES. The Ag loadings with L-ZIF-67-Ag-X (X = 0.1, 0.3 and 0.5) samples were 1.65, 3.99 and 6.28 wt%, respectively. The contents of Co with L-ZIF-67-Ag-X were determined to be 23.44, 22.89 and 20.11 wt%, respectively. Elemental analysis of L-ZIF-67-Ag-X (X = 0.1, 0.3 and 0.5) was investigated. Experimental results of L-ZIF-67-Ag-0.1: C 40.75 wt%, N 25.63 wt%, H 4.32 wt%. Experimental results of L-ZIF-67-Ag-0.3: C 39.82 wt%, N 25.01 wt%, H 4.21 wt%. Experimental results of L-ZIF-67-Ag-0.5: C 40.06 wt%, N 25.23 wt%, H 4.19 wt%.

Catalytic testing. In a typical experiment, 0.5 mmol of ethynylbenzene, 0.6 mmol of *tert*-butyl isocyanide, 0.1 mmol of PPh_3 (20 mol% relative to ethynylbenzene), 3 mL of CH_3CN and 50 mg activated catalyst L-ZIF-67-Ag-0.3 (The equivalence of silver was 3.69 mol% for each reaction.)

were added into 10 mL reaction tube. Subsequently, the reaction mixture was purged with O_2 for 3 times and stirred at 60 °C for 12 h with an oxygen balloon installed. After the catalytic reaction was complete, the heterogeneous catalyst was separated via filtration. The residual mixture was further purified by flash column chromatography on silica gel (petroleum ether/ethyl acetate = 20:1 as an eluent) to afford pure product.

Recyclability experiment. 1 mmol of ethynylbenzene, 1.2 mmol of *tert*-butyl isocyanide, 0.2 mmol of PPh_3 (20 mol% relative to ethynylbenzene), 6 mL of CH_3CN and 100 mg recovered catalyst L-ZIF-67-Ag-0.3 were put into a 25 mL Schlenk tube. The catalyst is filtered, washed in ethanol three times, and then dried in a vacuum. Finally, it was reused for the next round of catalytic reaction.

Activation reaction. No catalyst, $AgNO_3$, L-ZIF-67 and L-ZIF-67-Ag-0.3 systems were performed which were capped to O_2 or Ar balloon under the optimal reaction conditions. Subsequently, the mixture solution was stirred at 60 °C for 1 h and appropriate amount of supernatant were analyzed rapidly by ^{1}H NMR and ^{13}C NMR spectroscopy (DMSO-D_6).

FT-IR analysis.
1.5 mmol of ethynylbenzene, 1.8 mmol of *tert*-butyl isocyanide, 0.1 mmol of PPh_3, 3 mL of CH_3CN and 50 mg activated catalyst L-ZIF-67-Ag-0.3 were added into 10 mL reaction tube at 1 atm. oxygen atmosphere. A small amount reaction solution was collected per hour and rapidly freezed it at -20 °C (nitrogen atmosphere). The supernatant of the collected sample was tested immediately by FT-IR analysis.

Calculation details.
All calculations are carried out at a temperature of 333.15 K and all the density functional theory (DFT) calculations were carried out with the GAUSSIAN 09 series of programs.[2] DFT method B3LYP[3,4] with 6-31+G(d) basis set (SDD[5,6] basis set for Co) was used for geometry optimizations. Harmonic vibration frequency calculations were performed for all stationary points to confirm them as a local minima or transition structure and to derive the thermochemical corrections for the enthalpies and free energies. The solvent effects were considered by single point calculations on the gas-phase stationary points with a SMD continuum solvation model (acetonitrile as the solvent model).[7] M06-L with 6-311+G(d,p) basis set was used in the solvation single point energies to give more accurate energetic information.[8-10]

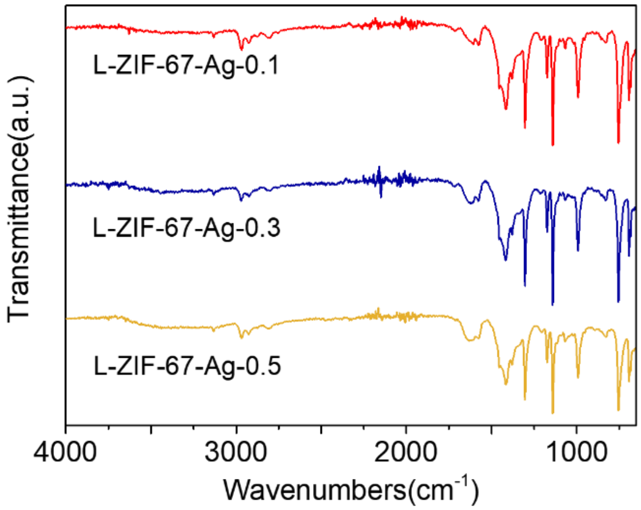

Figure S1 FI-IR Spectrum.

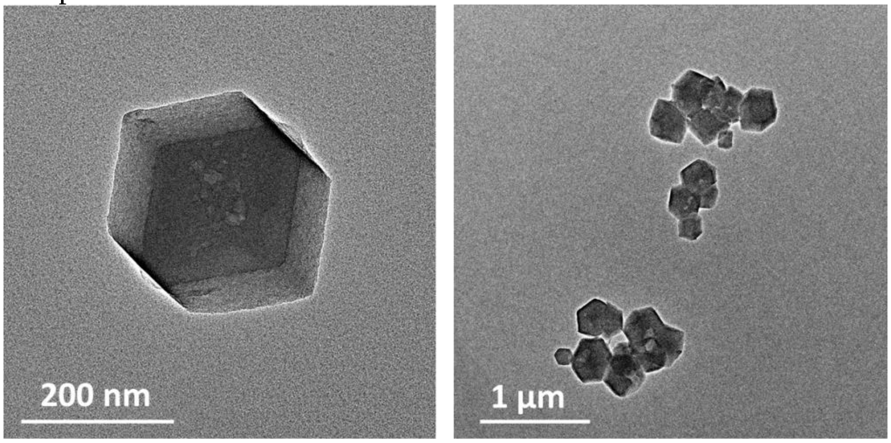

Figure S2 TEM images of L-ZIF-67.

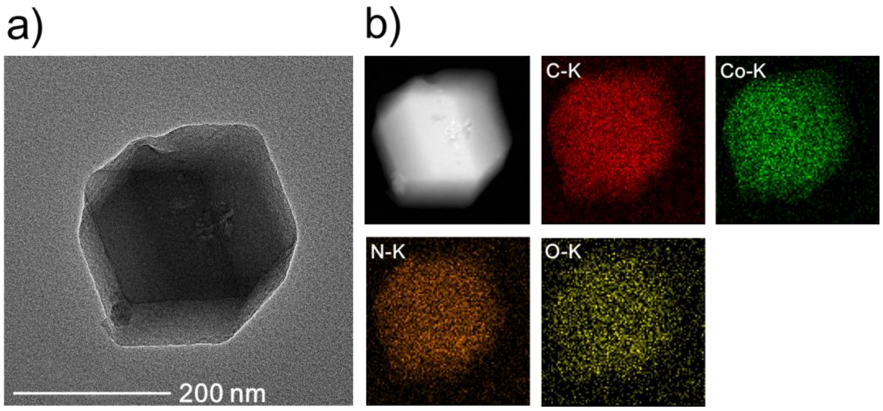

Figure S3 (a) TEM, (b) dark field STEM and elemental mapping images of L-ZIF-67.

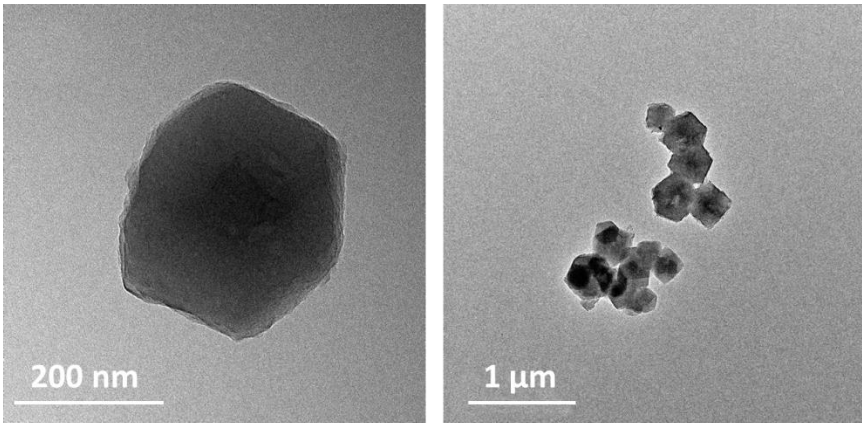

Figure S4 TEM images of the as-synthesized L-ZIF-67-Ag-0.3.

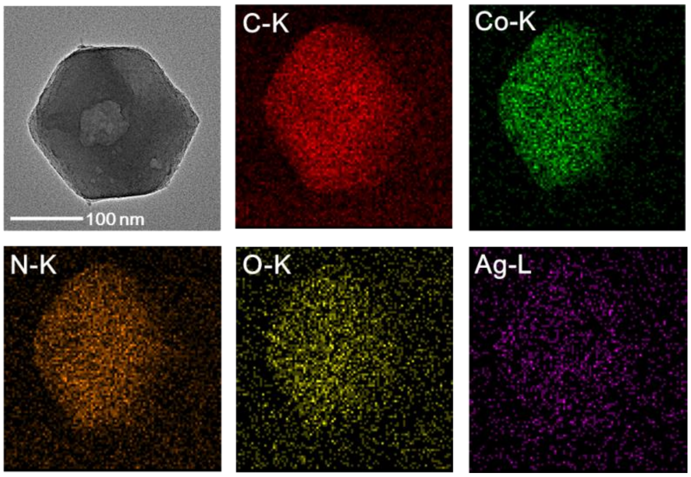

Figure S5 Elemental mapping images of L-ZIF-67-Ag-0.1.

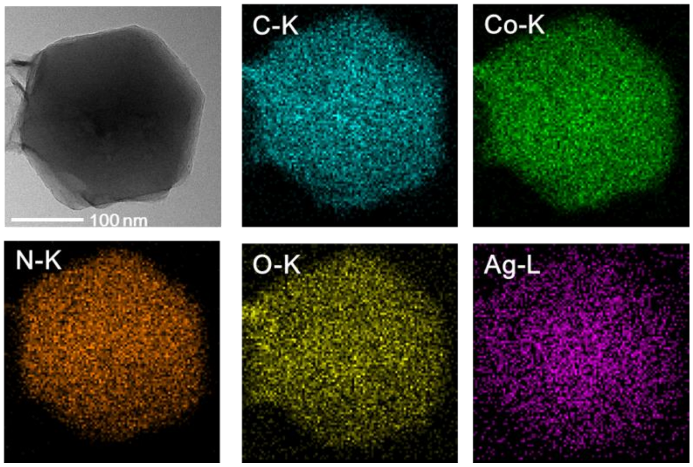

Figure S6 Elemental mapping images of L-ZIF-67-Ag-0.5.

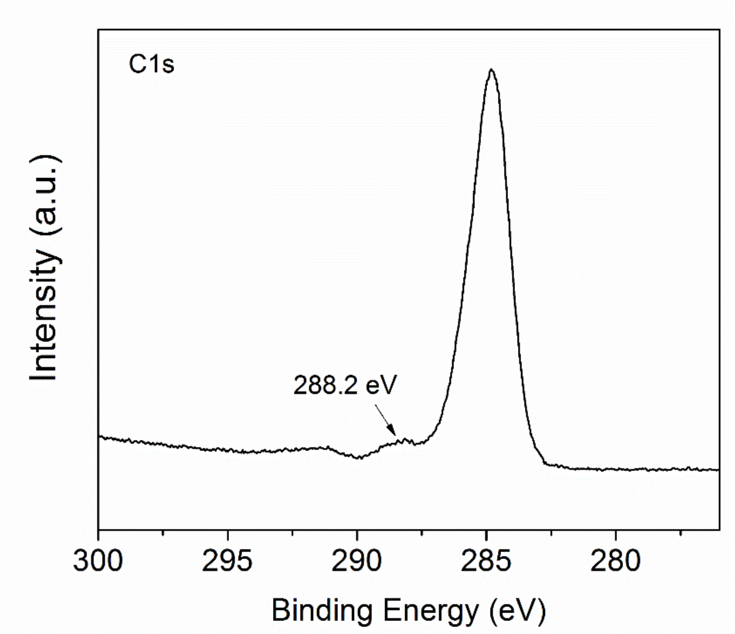

Figure S7 The XPS analysis of C1s spectrum of L-ZIF-67.

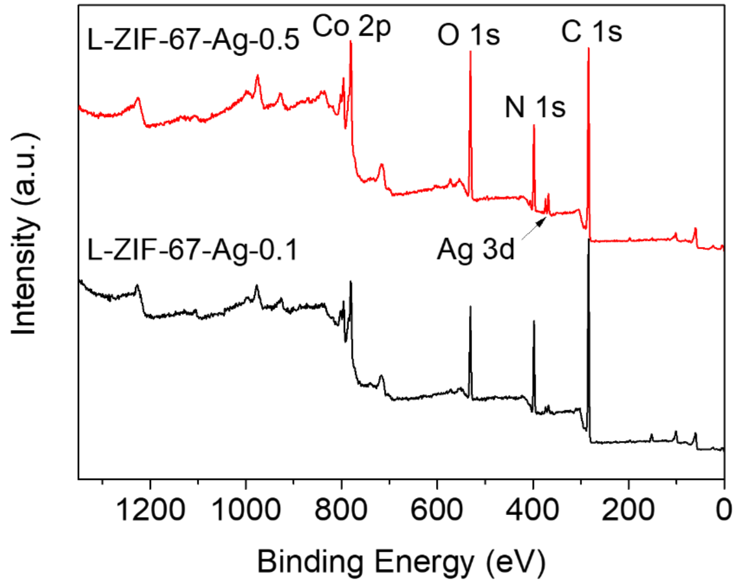

Figure S8 The XPS spectra of L-ZIF-67-Ag-0.1 and L-ZIF-67-Ag-0.5.

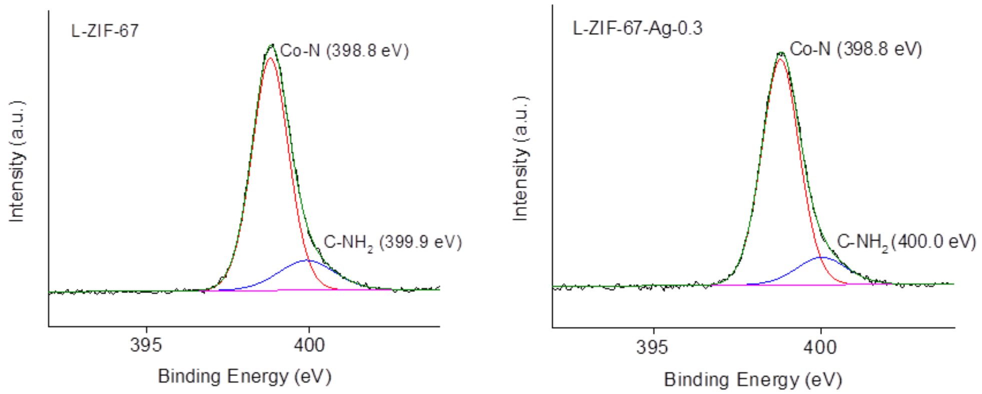

Figure S9 The XPS analysis of N1s spectrum of L-ZIF-67 and L-ZIF-67-Ag-0.3.

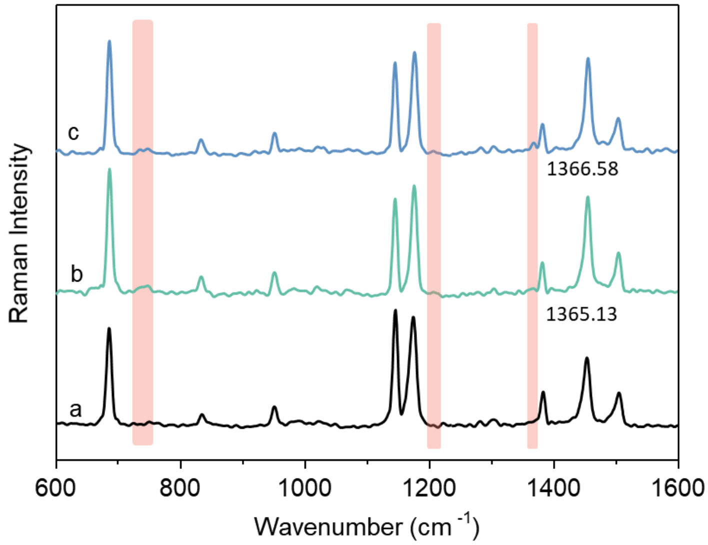

Figure S10 Raman spectra of (a) ZIF-67, (b) L-ZIF-67 and (c) L-ZIF-67-Ag-0.3 in the Raman shift range of 600-1600 cm^{-1}.

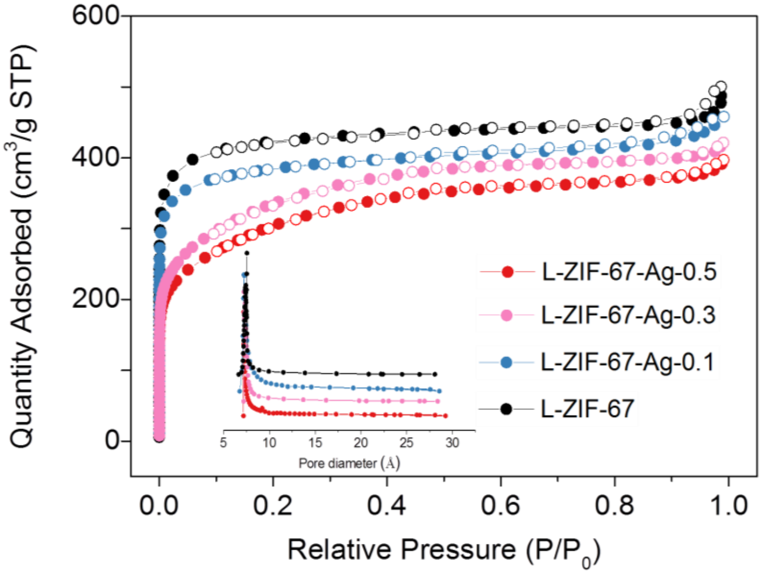

Figure S11 N_2 adsorption-desorption isotherms of L-ZIF-67, L-ZIF-67-Ag-0.1, L-ZIF-67-Ag-0.3 and L-ZIF-67-Ag-0.5. Inset: pore-size distributions for the four MOFs determined from their N_2 adsorption isotherms at 77 K.

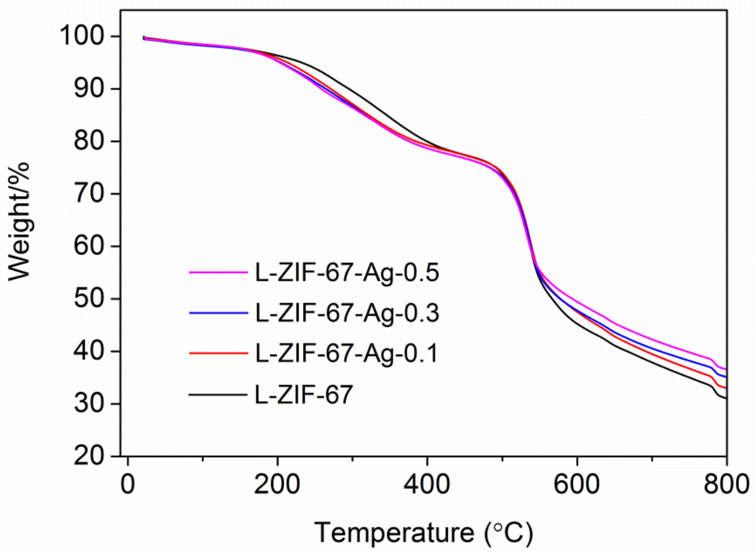

Figure S12 TGA diagrams of L-ZIF-67, L-ZIF-67-Ag-0.1, L-ZIF-67-Ag-0.3 and L-ZIF-67-Ag-0.5.

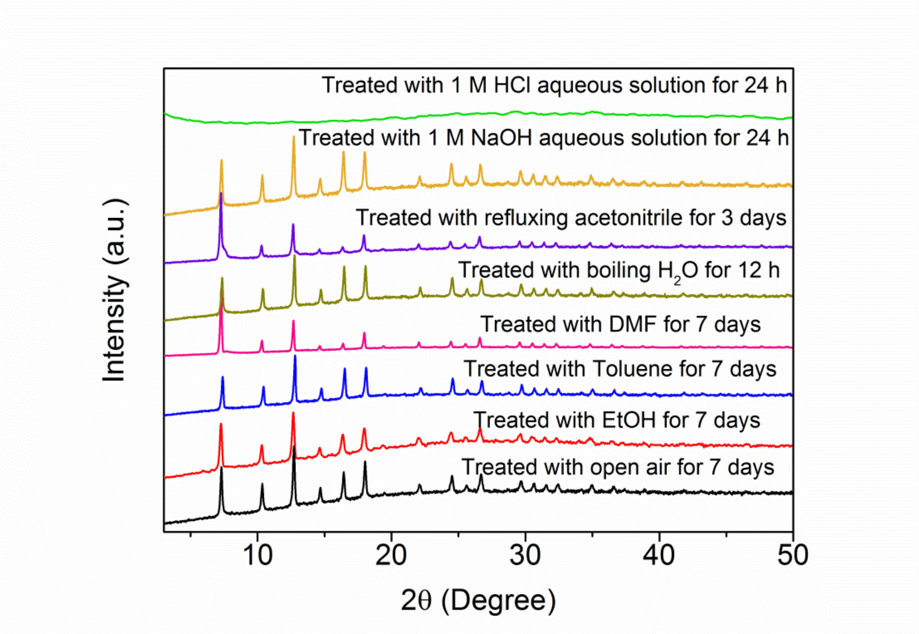

Figure S13 PXRD pattern of L-ZIF-67-Ag-0.3 samples after different treatments.

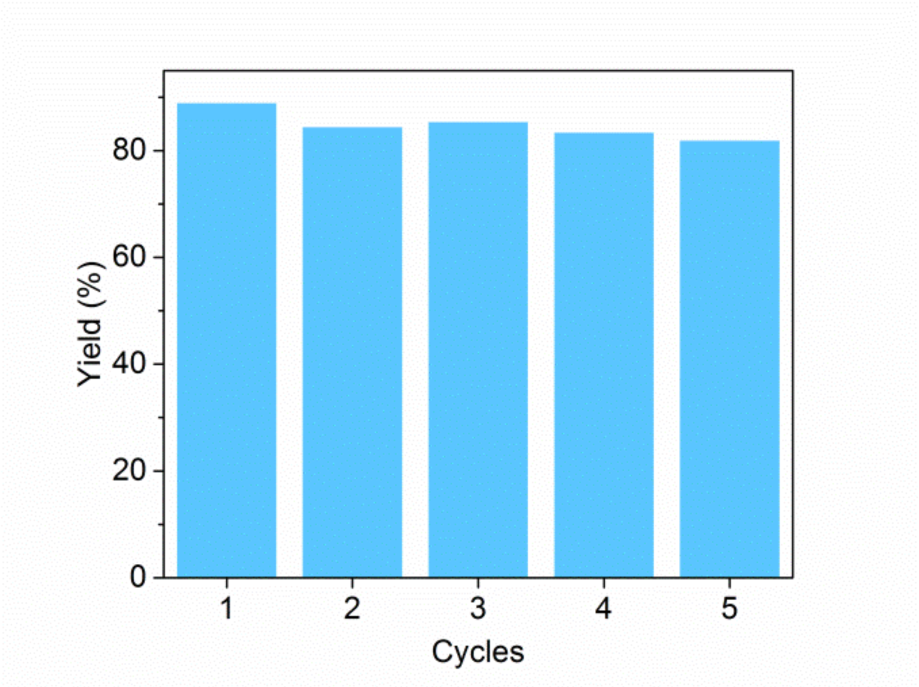

Figure S14 Recycling stability of L-ZIF-67-Ag-0.3 for the synthesis of alkynamide between ethynylbenzene, tert-butyl isocyanide and O_2.

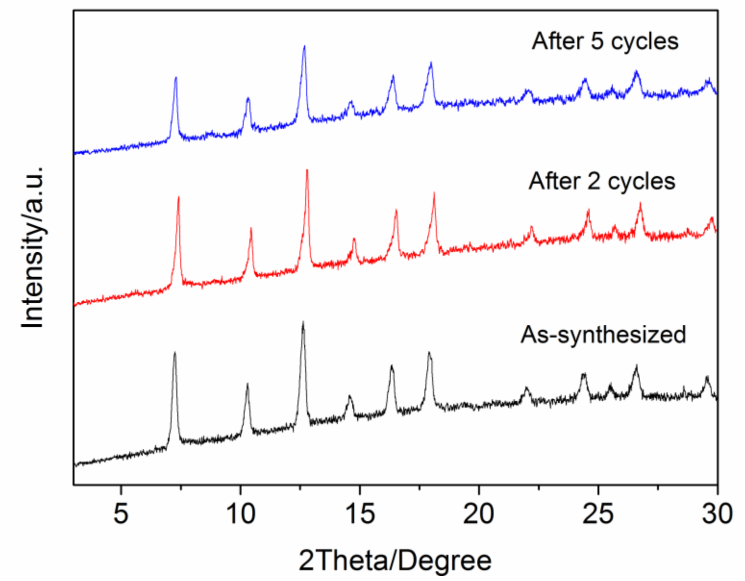

Figure S15 PXRD pattern of L-ZIF-67-Ag-0.3: before and after catalytic reaction.

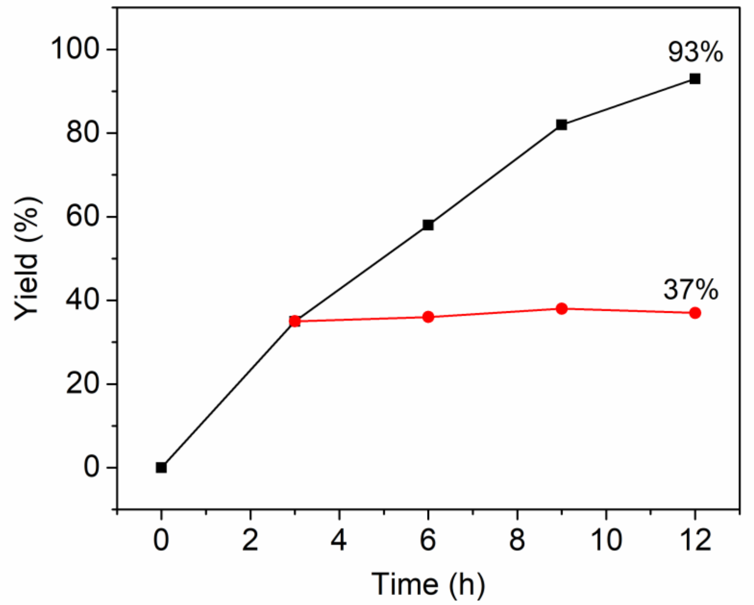

Figure S16 Black: time-dependent kinetic curves for for the synthesis of alkynamide between ethynylbenzene, tert-butyl isocyanide and O_2, Red: the L-ZIF-67/0.3Ag catalyst removed at 3h.

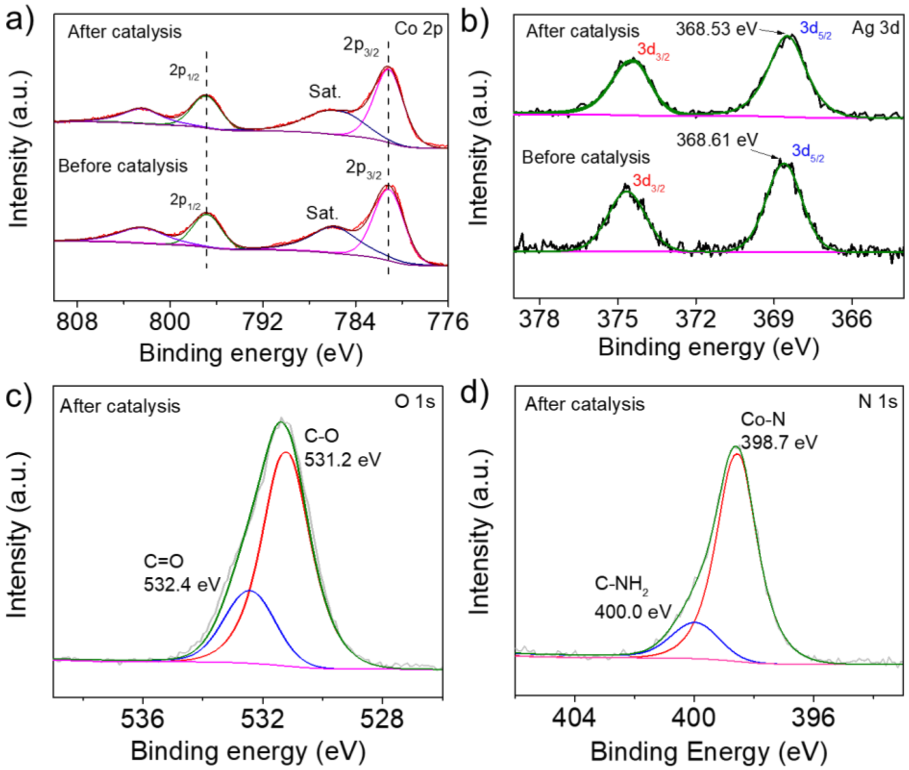

Figure S17 XPS analysis of (a) Co 2p, (b) Ag 3d, (c) N 1s and (d) O1s regions for the L-ZIF-67-Ag-0.3 after catalytic reactions.

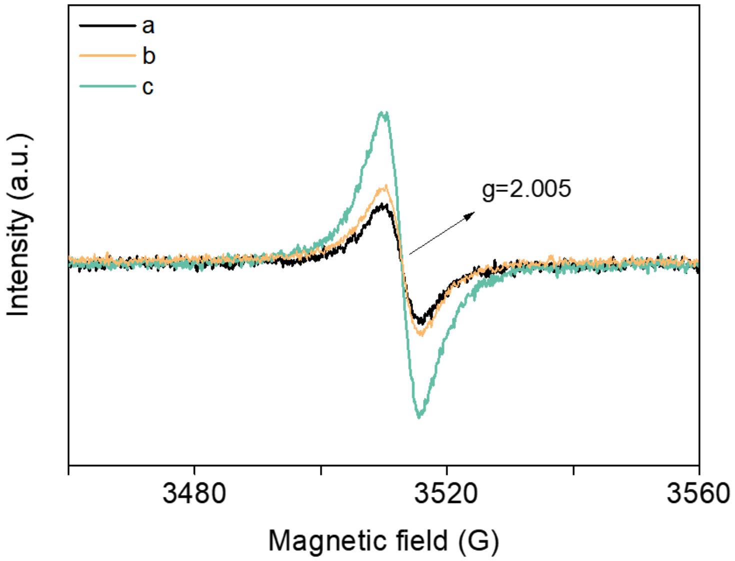

Figure S18 EPR spectra of (a) L-ZIF-67, (b) L-ZIF-67-Ag-0.3 and (c) L-ZIF-67-Ag-0.3 after the catalytic reaction.

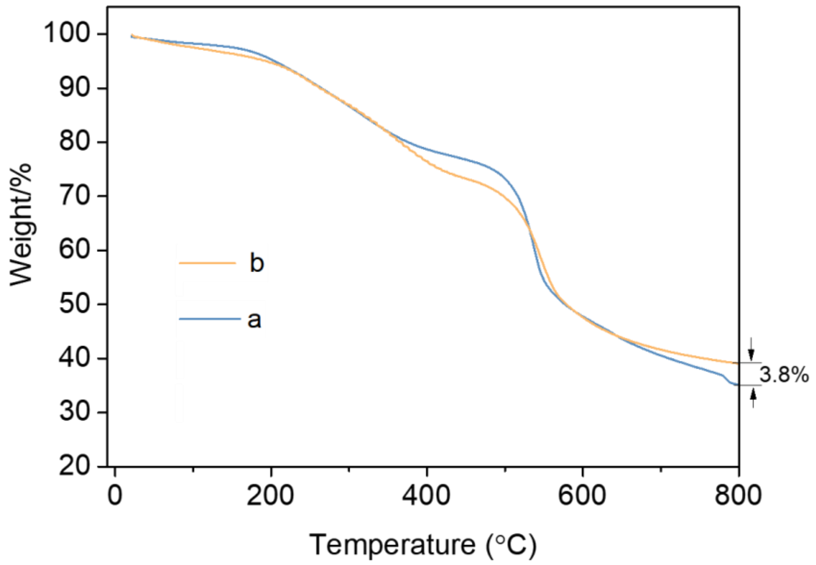

Figure S19 TGA diagrams of (a) L-ZIF-67-Ag-0.3 and (b) L-ZIF-67-Ag-0.3 after the catalytic reaction.

Table S1 Catalytic performance of various catalytic systems in aminocarbonylation of terminal alkynes.

Catalyst	Mol%	Carbonyl sources	Time (h)	Temp (°C)	Yield (%)	TOF (h^{-1})[a]
$Mo(CO)_6$	100	$Mo(CO)_6$	2	110	83[11]	0.42
$PdCl_2(PPh_3)_2$	5	CO	0.83	80	60[12]	14.5
$PdCl_2$	2	CO	24	rt	78[13]	1.6
$Pd(OAc)_2$	0.2	CO	6	80	87[14]	72.5
$Pd(OAc)_2$	1	CO	20	110	99[15]	4.9
Pd/C	10	CO	14	80	96[16]	0.68
$PdCl_2(PPh_3)_2$	2.5	CO	20	50	70[17]	5.44
$Fe_3(CO)_{12}$	5	CO	15	120	89[18]	1.18
$Pd_2(dba)_3$	3.3	$Co_2(CO)_8$	5	rt	92[19]	5.57
Pd(0)	2.5	CO	16	100	88[20]	2.2
Ir/$NiCl_2$	15	CO	0.67	rt	81[21]	8.1
This work	3.69 (42.4)	Isocyanides	12	60	93	2.1[b] (0.18)[c]

[a]TOF: Turnover frequency was calculated by the mole number of products formed per mole number of catalytic sites per hour and evaluated under the optimal conditions. [b]L-ZIF-67-Ag-0.3 (The number of moles of Ag sites). [c]L-ZIF-67-Ag-0.3 (The number of moles of Ag and Co sites).

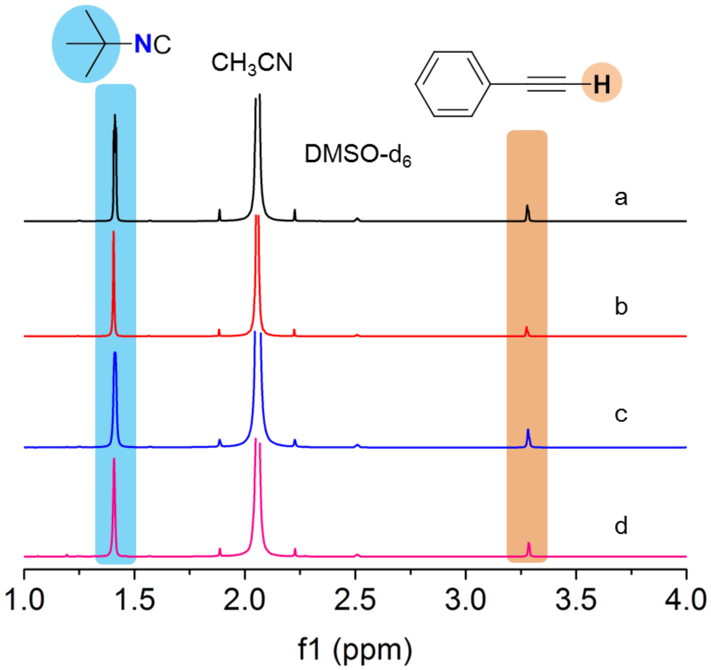

Figure S20 ^{1}H NMR spectra changes on attempting activation of terminal alkynes by no catalyst (a), $AgNO_3$ (b), L-ZIF-67 (c) and L-ZIF-67-Ag-0.3 (d) systems at 1 atm. Ar atmosphere (in DMSO-D_6).

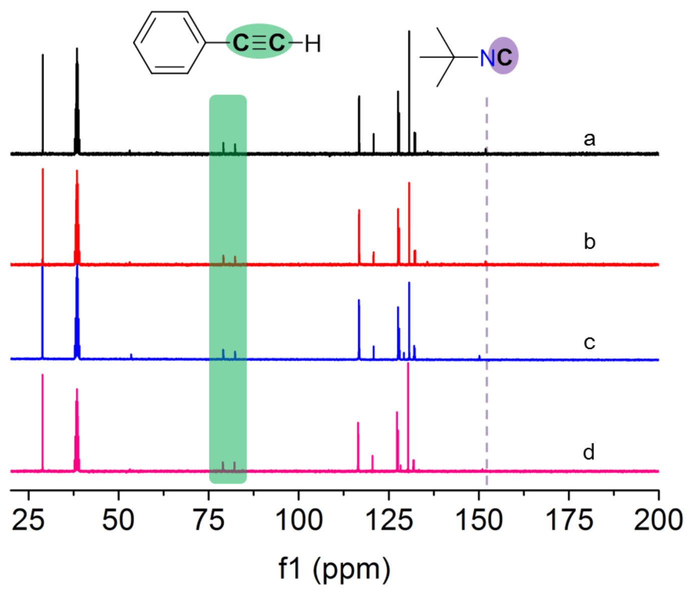

Figure S21 ^{13}C NMR spectra changes on attempting activation of terminal alkynes by no catalyst (a), $AgNO_3$ (b), L-ZIF-67 (c) and L-ZIF-67-Ag-0.3 (d) systems at 1 atm. Ar atmosphere (in DMSO-D_6).

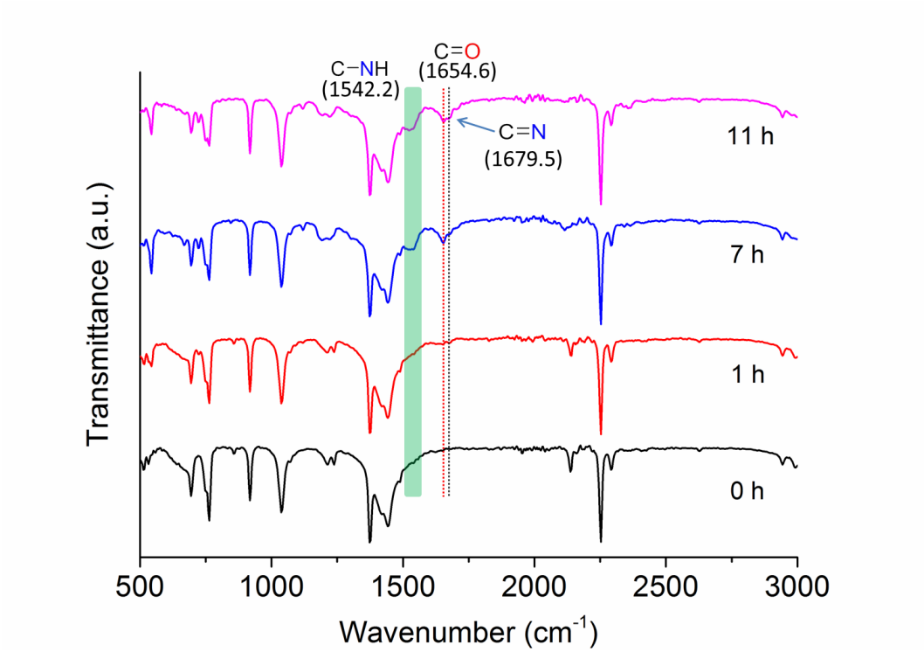

Figure S22 The continuous monitoring of FT-IR spectra at 0 h, 1h, 7h and 11 h.

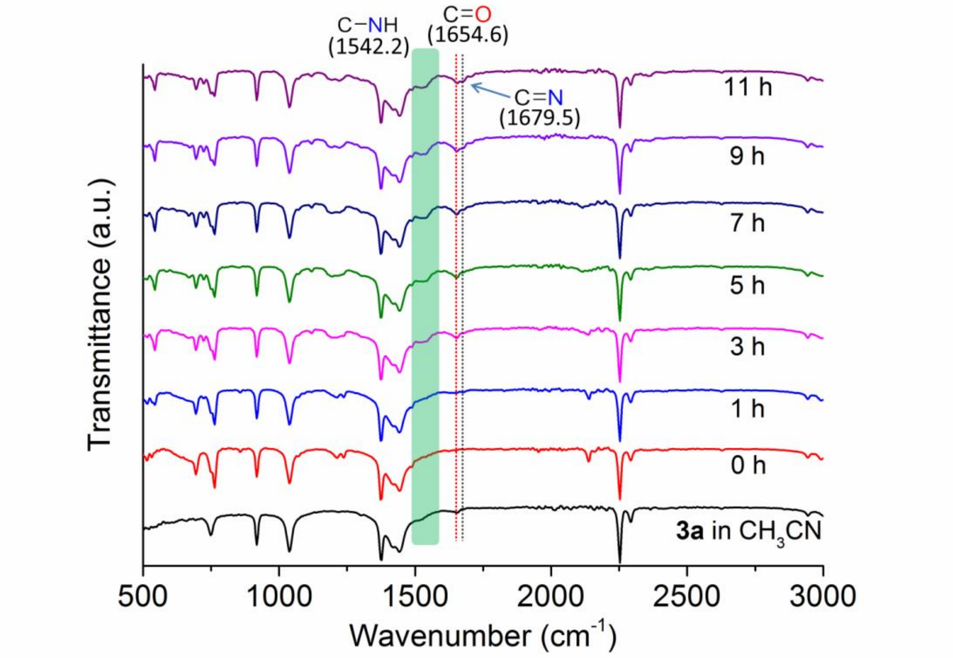

Figure S23 The continuous monitoring of FT-IR spectra at 0 h, 1h, 2h, 3h, 5h, 7h, 9h and 11 h.

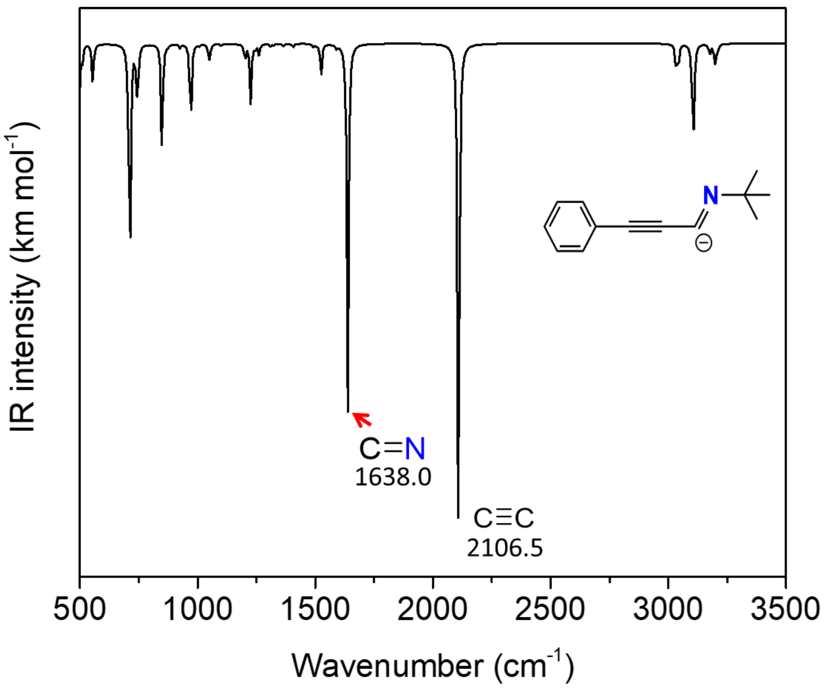

Figure S24 The simulated FT-IR spectrum of the intermediate product **F**.

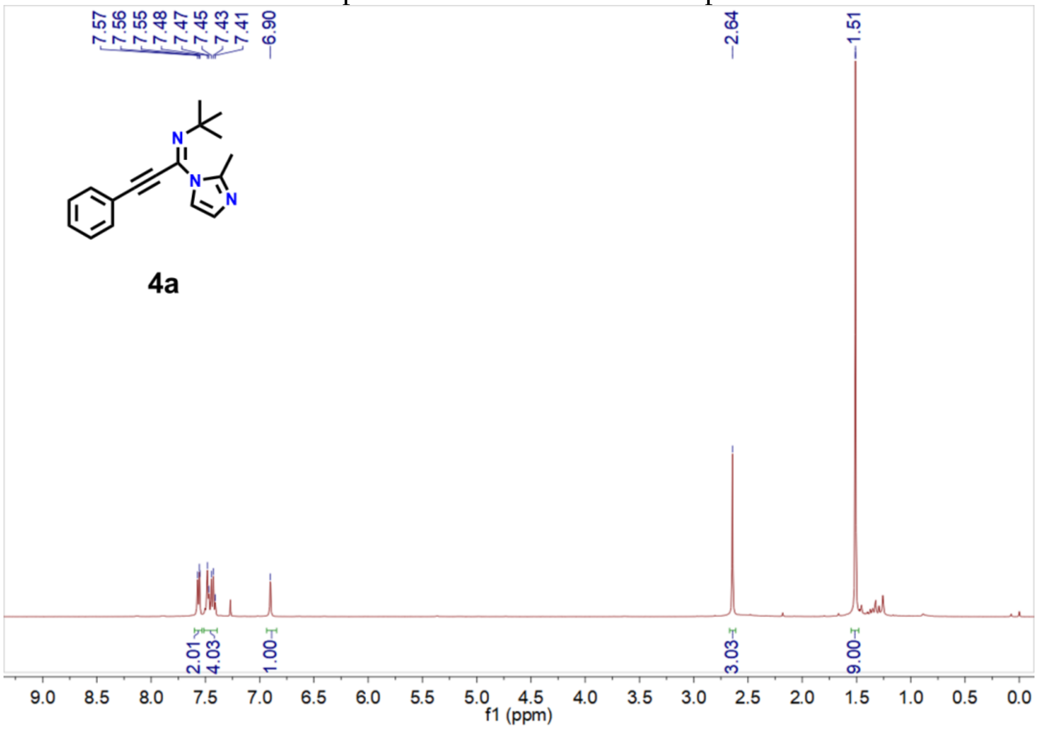

Figure S25 ^{1}H NMR (400 MHz, $CDCl_3$) of compound **4a**.

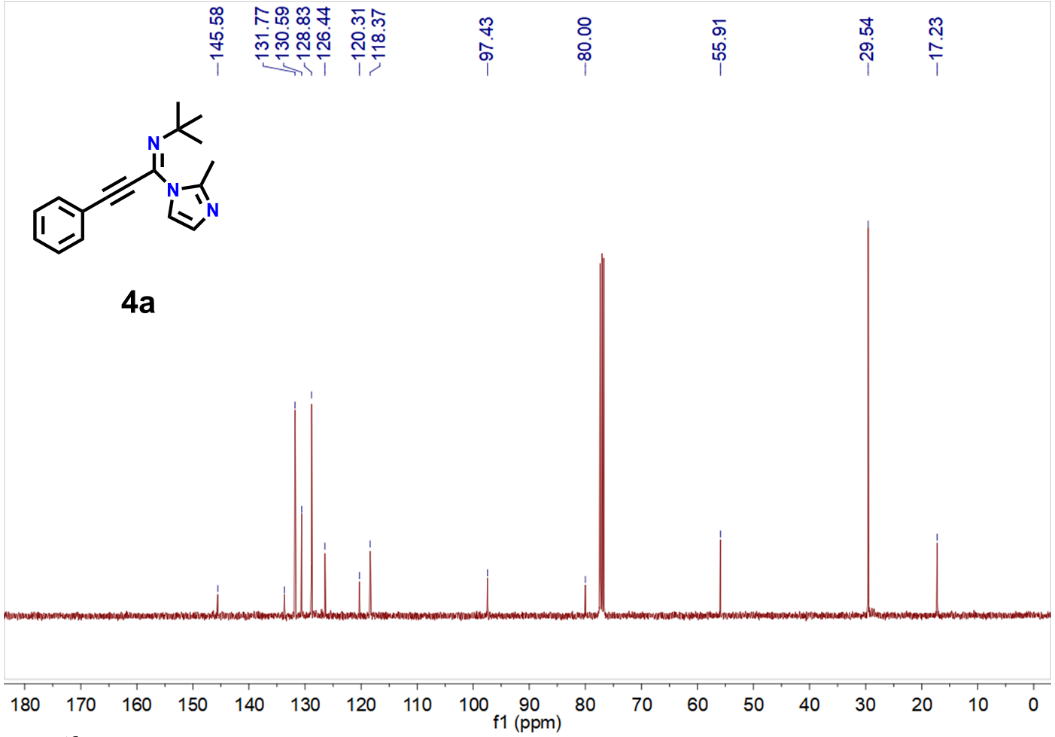

Figure S26 ^{13}C NMR (101 MHz, $CDCl_3$) of compound **4a**.

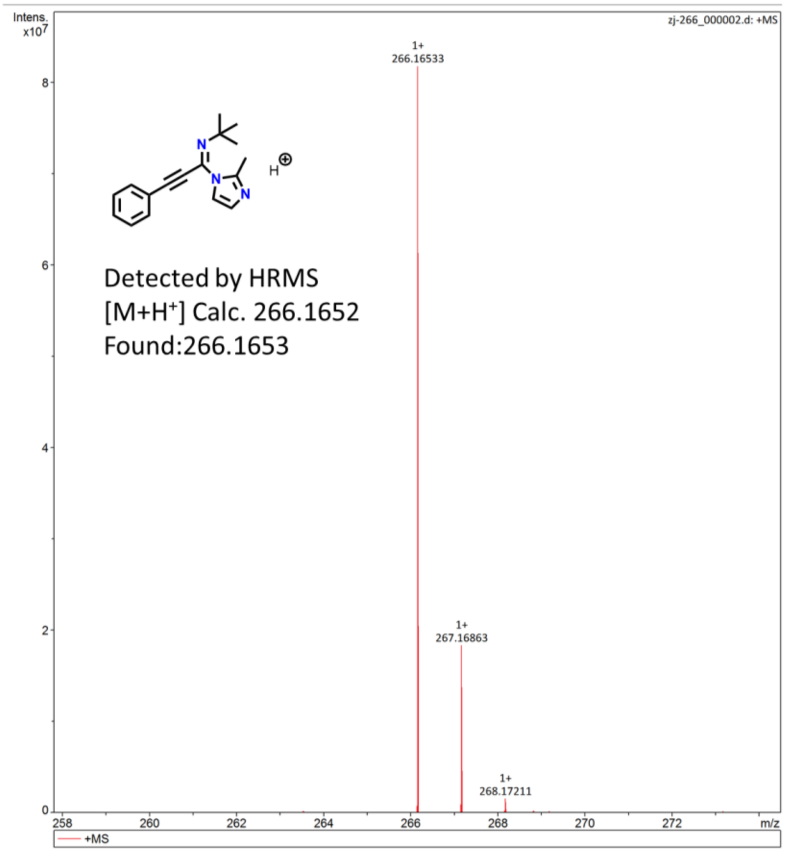

Figure S27 Mass spectrometry of compound **4a**.

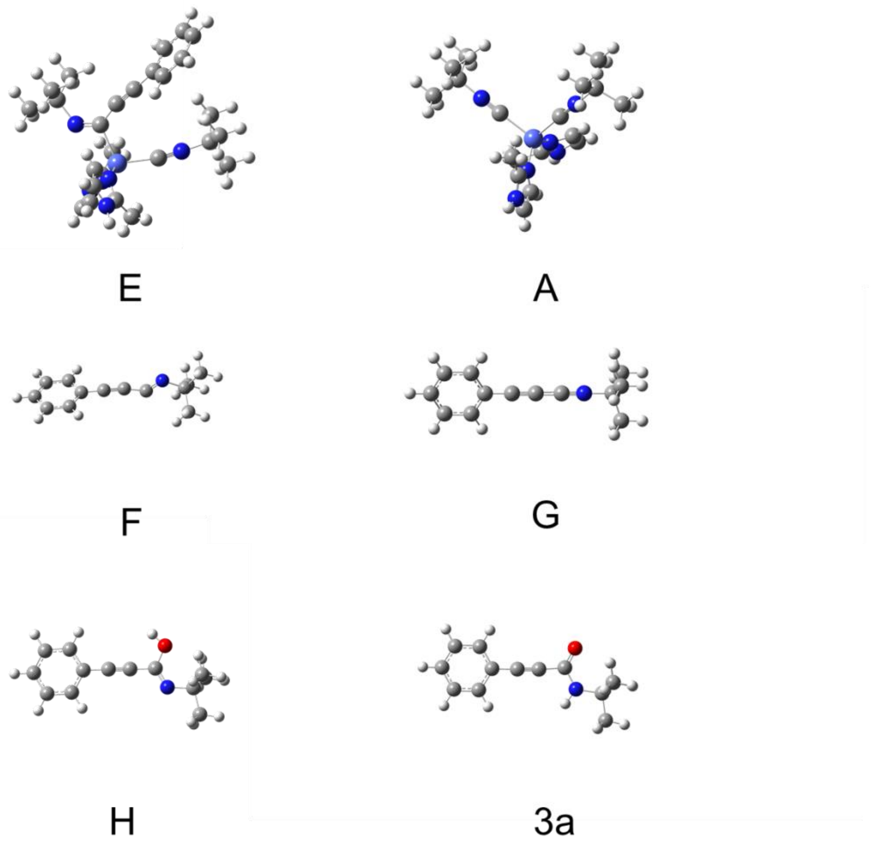

Figure S28 Optimized structure of possible intermediate **A, E-H** and **3a**.

Spectroscopic Data of Compounds[22,23]

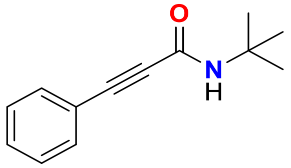

N-(tert-butyl)-3-phenylpropiolamide (3a): White solid, isolated yield, 93%. **^{1}H NMR** (400 MHz, $CDCl_3$) δ 7.52-7.48 (m, 2H), 7.40-7.36 (m, 1H), 7.34-7.31 (m, 2H), 5.88 (s, 1H), 1.41 (s, 9H), **^{13}C NMR** (101 MHz, $CDCl_3$) δ 152.65, 132.41, 129.85, 128.48, 120.44, 84.07, 82.63, 52.45, 28.63.

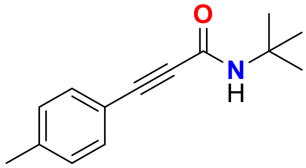

N-(tert-butyl)-3-(p-tolyl)propiolamide (3b): White solid, isolated yield, 89% **^{1}H NMR** (400 MHz, $CDCl_3$) δ 7.41 (d, *J* = 8.0 Hz, 2H), 7.15 (d, *J* = 8.0 Hz, 2H), 5.74 (s, 1H), 2.36 (s, 3H), 1.41

(s, 9H). **^{13}C NMR** (101 MHz, $CDCl_3$) δ 152.74, 140.32, 132.37, 129.27, 117.31, 83.65, 82.93, 52.39, 28.66, 21.64.

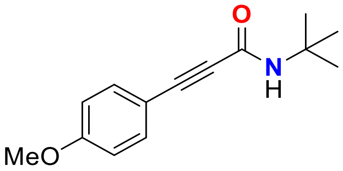

N-(tert-butyl)-3-(4-methoxyphenyl)propiolamide (3c): White solid, isolated yield, 85%. **^{1}H NMR** (400 MHz, $CDCl_3$) δ 7.46 (d, *J* = 7.2 Hz, 2H), 6.86 (d, *J* = 7.2 Hz, 2H), 5.72 (s, 1H), 3.82 (s, 3H), 1.41 (s, 9H). **^{13}C NMR** (101 MHz, $CDCl_3$) δ 160.82, 152.90, 134.16, 114.17, 112.28, 83.28, 83.04, 55.36, 52.35, 28.68.

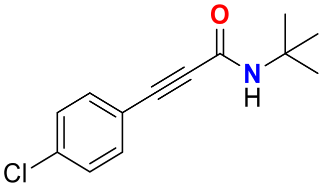

N-(tert-butyl)-3-(4-chlorophenyl)propiolamide (3d): White solid, isolated yield, 87%. **^{1}H NMR** (400 MHz, $CDCl_3$) δ 7.46 (d, *J* = 8.4 Hz, 2H), 7.34 (d, *J* = 8.4 Hz, 2H), 5.74 (s, 1H), 1.41 (s, 9H). **^{13}C NMR** (101 MHz, $CDCl_3$) δ 152.26, 136.16, 133.60, 128.94, 118.93, 84.85, 81.33, 52.53, 28.64.

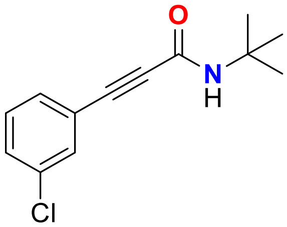

N-(tert-butyl)-3-(3-chlorophenyl)propiolamide (3e): White solid, isolated yield, 81%. **^{1}H NMR** (400 MHz, $CDCl_3$) δ 7.52-7.29 (m, 4H), 5.76 (s, 1H), 1.41 (s, 9H). **^{13}C NMR** (101 MHz, $CDCl_3$) δ 152.11, 134.38, 132.15, 130.53, 130.18, 129.79, 122.19, 84.85, 80.83, 52.58, 28.63.

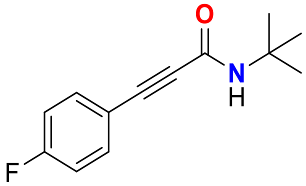

N-(tert-butyl)-3-(4-fluorophenyl)propiolamide (3f): White solid, isolated yield, 75%. **^{1}H NMR** (400 MHz, $CDCl_3$) δ 7.53-7.49 (m, 2H), 7.11-7.02 (m, 2H), 5.74 (s, 1H), 1.41 (s, 9H). **^{13}C NMR** (101 MHz, $CDCl_3$) δ 164.67, 162.16, 152.43, 134.58, 134.50, 116.54, 116.50, 116.08, 115.86, 83.86, 81.54, 52.49, 28.64.

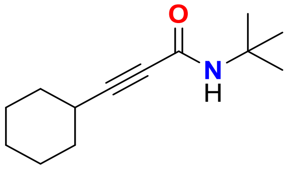

N-(tert-butyl)-3-cyclohexylpropiolamide (3g): Yellow solid, isolated yield, 72%. **^{1}H NMR** (400 MHz, $CDCl_3$) δ 5.55 (s, 1H), 2.42 (tt, *J* = 12 Hz, 1H), 1.80 (d, *J* = 12 Hz, 2H), 1.69 (d, *J* = 12 Hz,

2H), 1.49-1.38 (m, 4H), 1.36 (s, 9H), 1.34-1.30 (m, 2H). **^{13}C NMR** (101 MHz, $CDCl_3$) δ 152.92, 88.64, 76.39, 52.08, 31.77, 28.77, 28.62, 28.27, 25.65, 24.78.

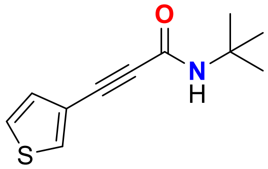

N-(tert-butyl)-3-(thiophen-3-yl)propiolamide (3h): Yellow solid, isolated yield, 63%. **^{1}H NMR** (400 MHz, $CDCl_3$) δ 7.62-7.16 (m, 3H), 5.78 (s, 1H), 1.41 (s, 9H). **^{13}C NMR** (101 MHz, $CDCl_3$) δ 152.61, 131.99, 129.94, 125.89, 119.57, 83.92, 78.04, 52.45, 28.65.

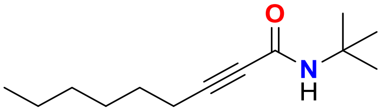

N-(tert-butyl)non-2-ynamide(3i): Yellow oil, isolated yield, 55%. **^{1}H NMR** (400 MHz, $CDCl_3$) δ 5.57 (s, 1H), 2.25 (t, *J* =8 Hz, 2H), 1.57-1.50 (m, 2H), 1.36 (s, 9H), 1.32-1.22 (m, 6H), 0.88 (t, *J* =8 Hz, 3H). **^{13}C NMR** (101 MHz, $CDCl_3$) δ 152.78, 85.19, 76.53, 52.08, 31.26, 28.61, 28.30, 27.78, 22.49, 18.51, 14.05.

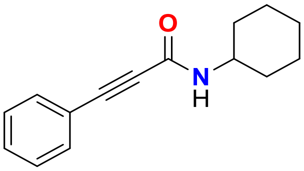

N-cyclohexyl-3-phenylpropiolamide(3j): Yellow solid, isolated yield, 64%. **^{1}H NMR** (400 MHz, $CDCl_3$) δ7.56-7.52 (m, 2H), 7.44-7.33 (m, 3H), 5.84 (s, 1H), 3.92-3.83 (m, 1H), 2.01-1.97 (m, 2H), 1.77-1.71 (m, 2H), 1.65-1.62 (m, 2H), 1.42-1.33 (m, 2H), 1.22-1.17 (m, 2H). **^{13}C NMR** (101 MHz, $CDCl_3$) δ 152.53, 132.46, 129.96, 128.59, 128.51, 120.35, 84.19, 83.36, 48.86, 32.88, 25.43, 24.77.

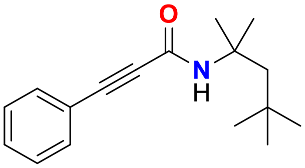

3-phenyl-N-(2,4,4-trimethylpentan-2-yl)ropiolamide (3k): Yellow solid, isolated yield, 53%. **^{1}H NMR** (400 MHz, $CDCl_3$) δ 7.53-7.33 (m, 5H), 5.71 (s, 1H), 1.80 (s, 2H), 1.46 (s, 6H), 1.04 (s, 9H). **^{13}C NMR** (101 MHz, $CDCl_3$) δ 152.48, 132.45, 129.83, 128.46, 120.46, 84.18, 82.40, 77.23, 56.36, 31.72, 31.44, 29.15.

Scanned ^{1}H NMR, and ^{13}C NMR spectra of compounds

^{1}H NMR of compound 3a

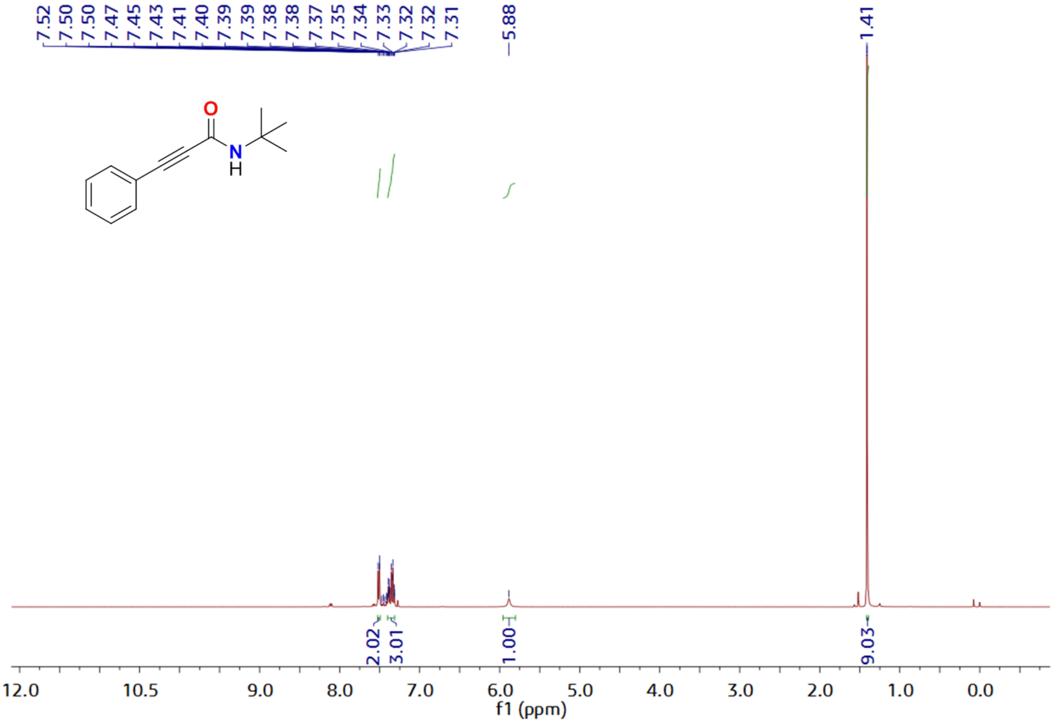

^{13}C NMR of compound 3a

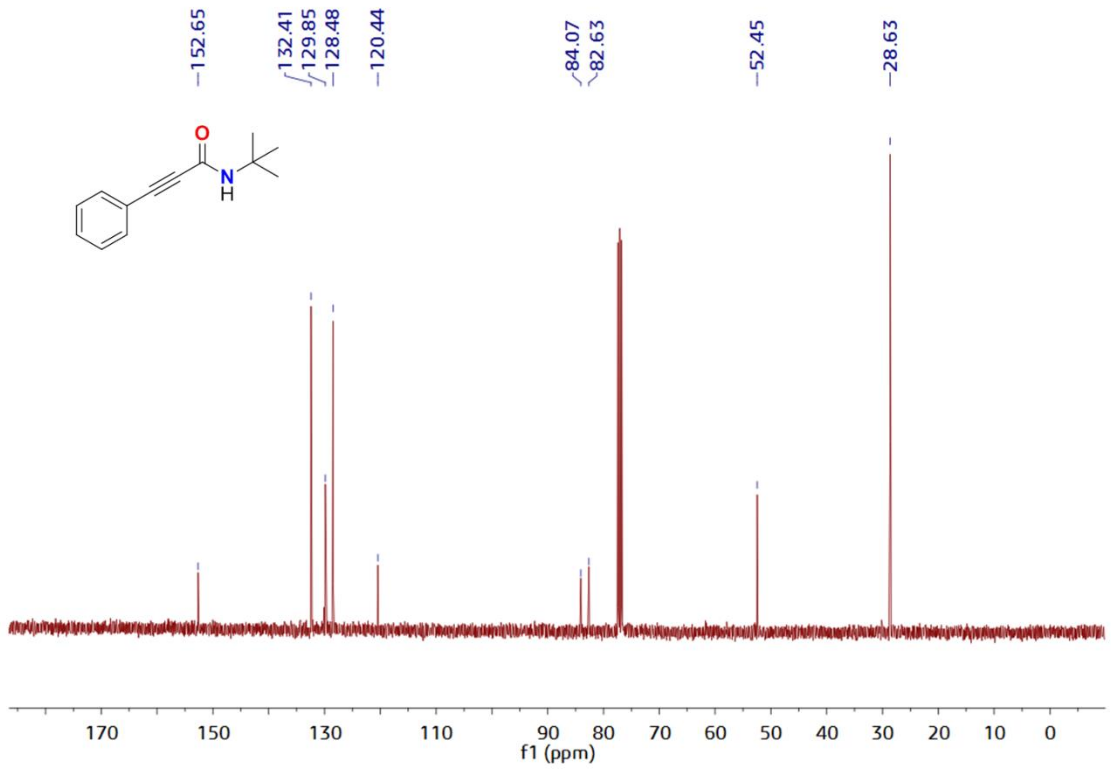

^{1}H NMR of compound 3b

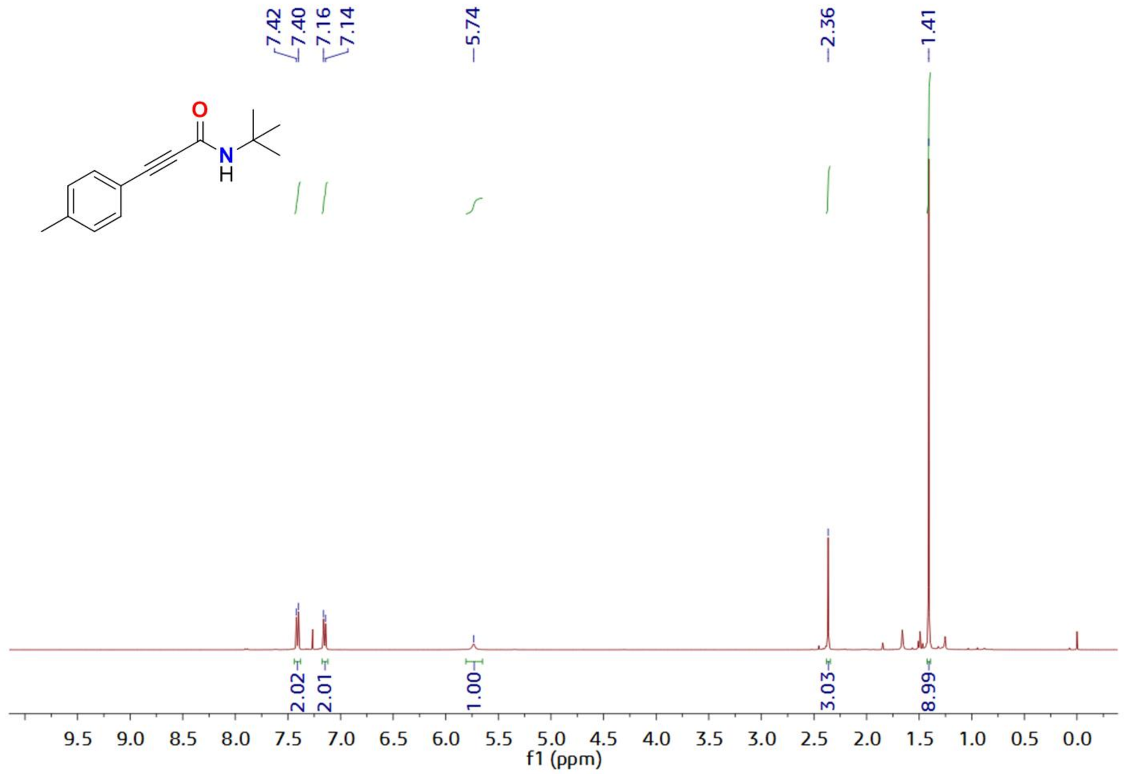

^{13}C NMR of compound 3b

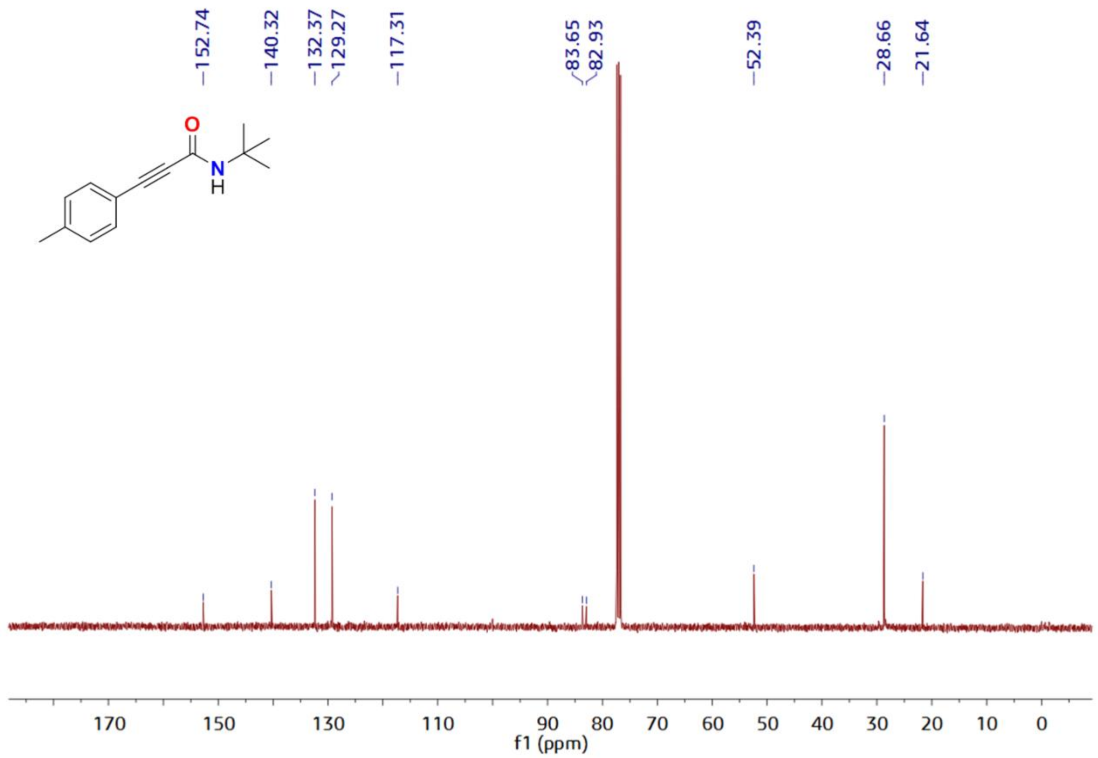

^{1}H NMR of compound 3c

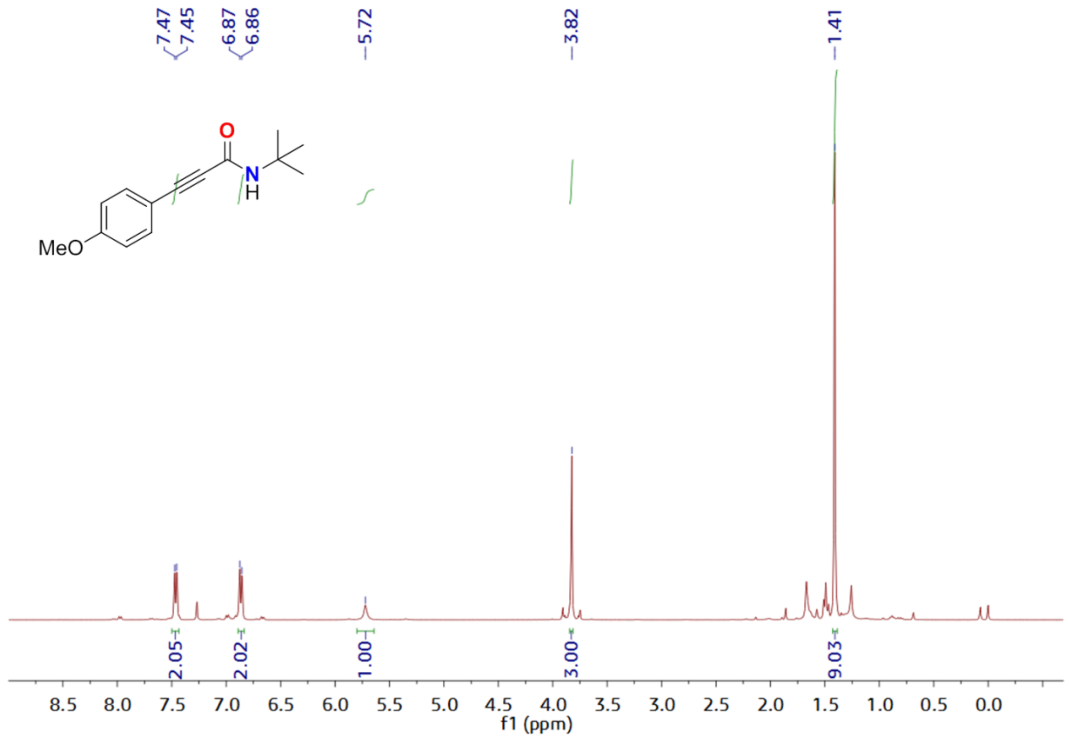

^{13}C NMR of compound 3c

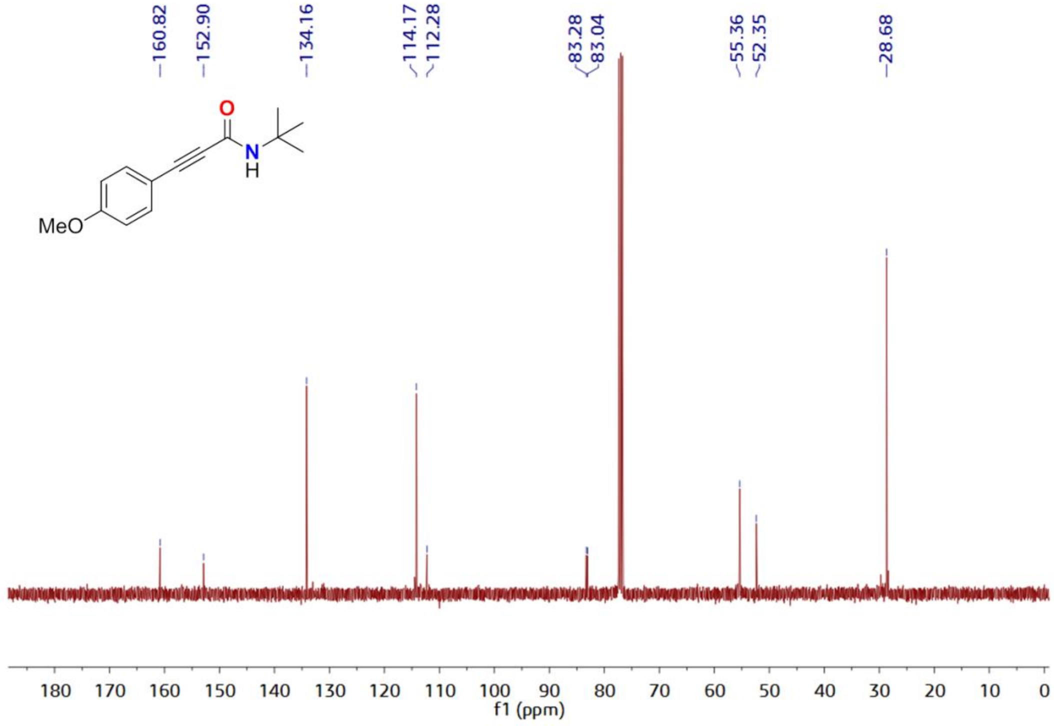

¹H NMR of compound 3d

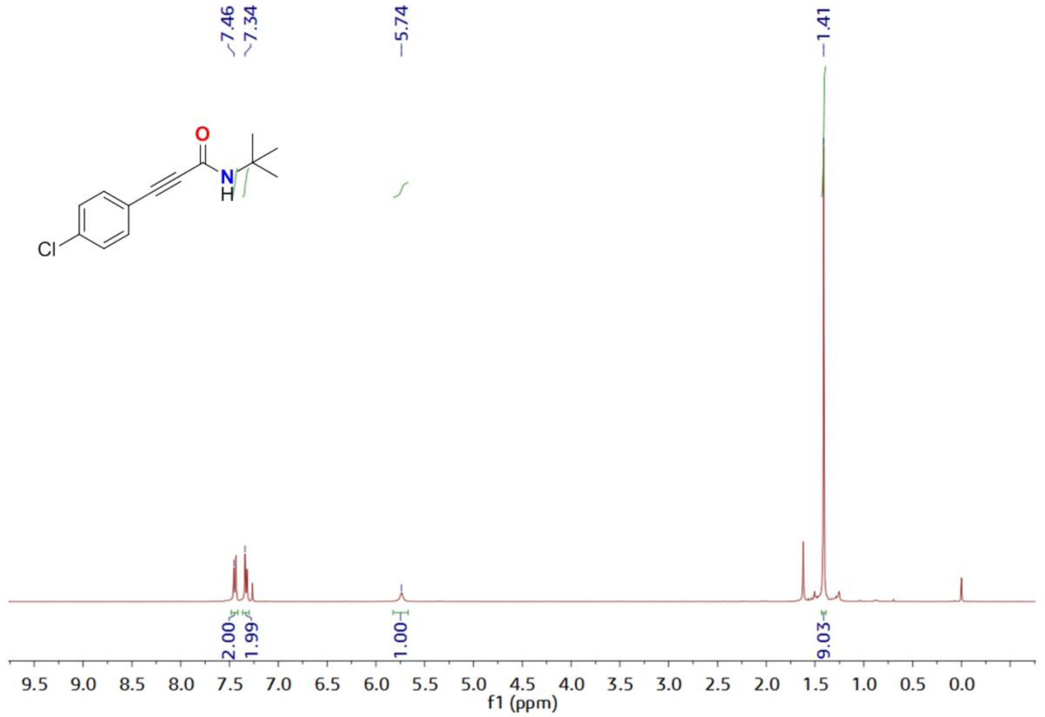

¹³C NMR of compound 3d

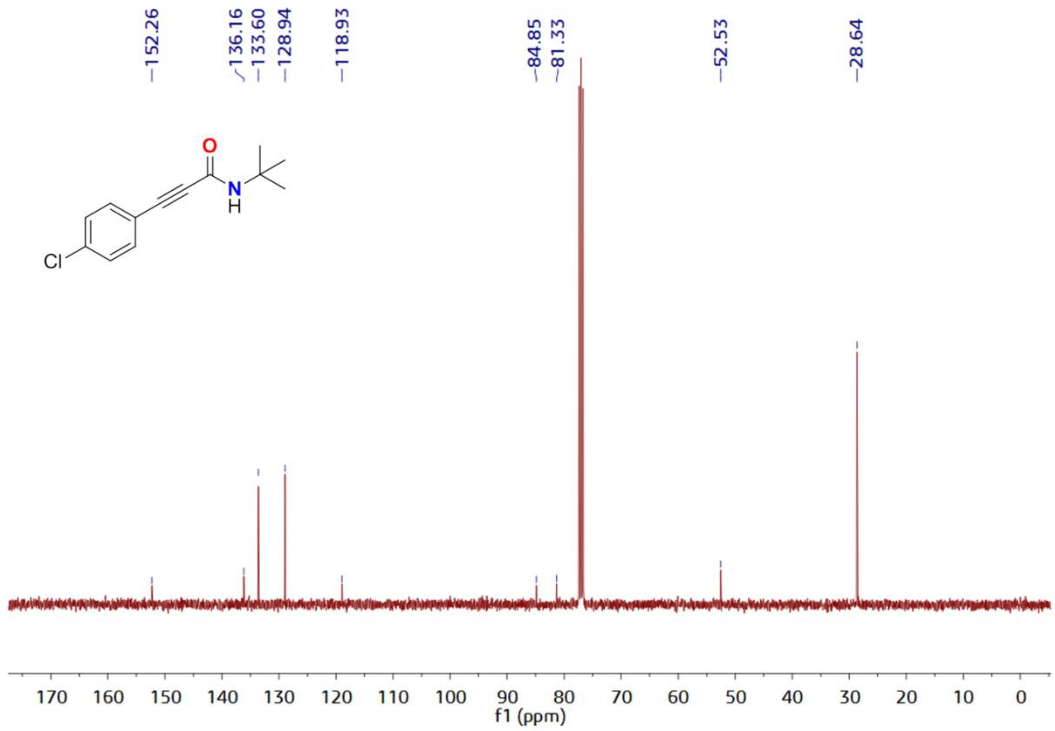

^{1}H NMR of compound 3e

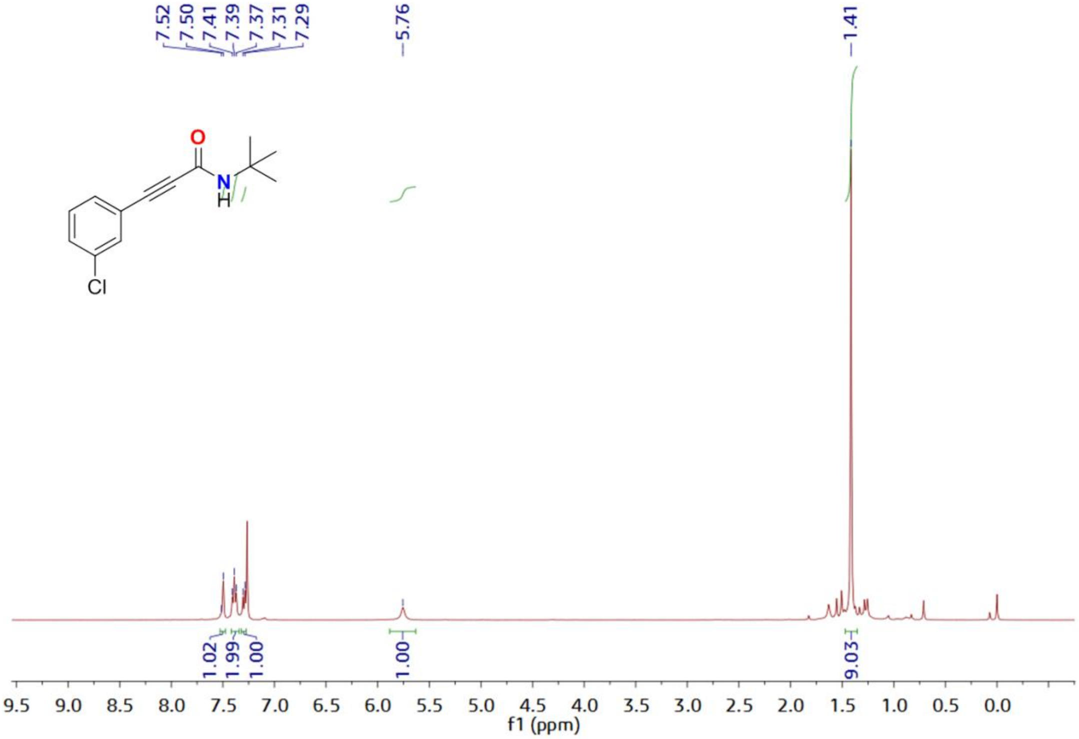

^{13}C NMR of compound 3e

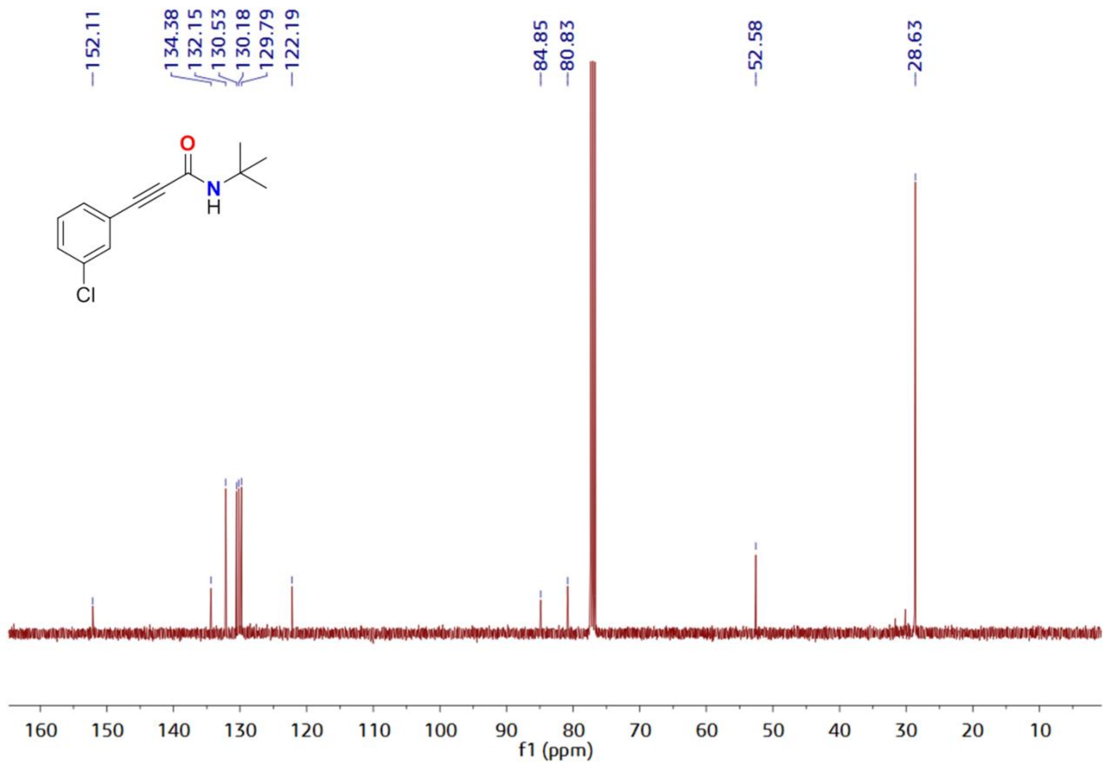

^{1}H NMR of compound 3f

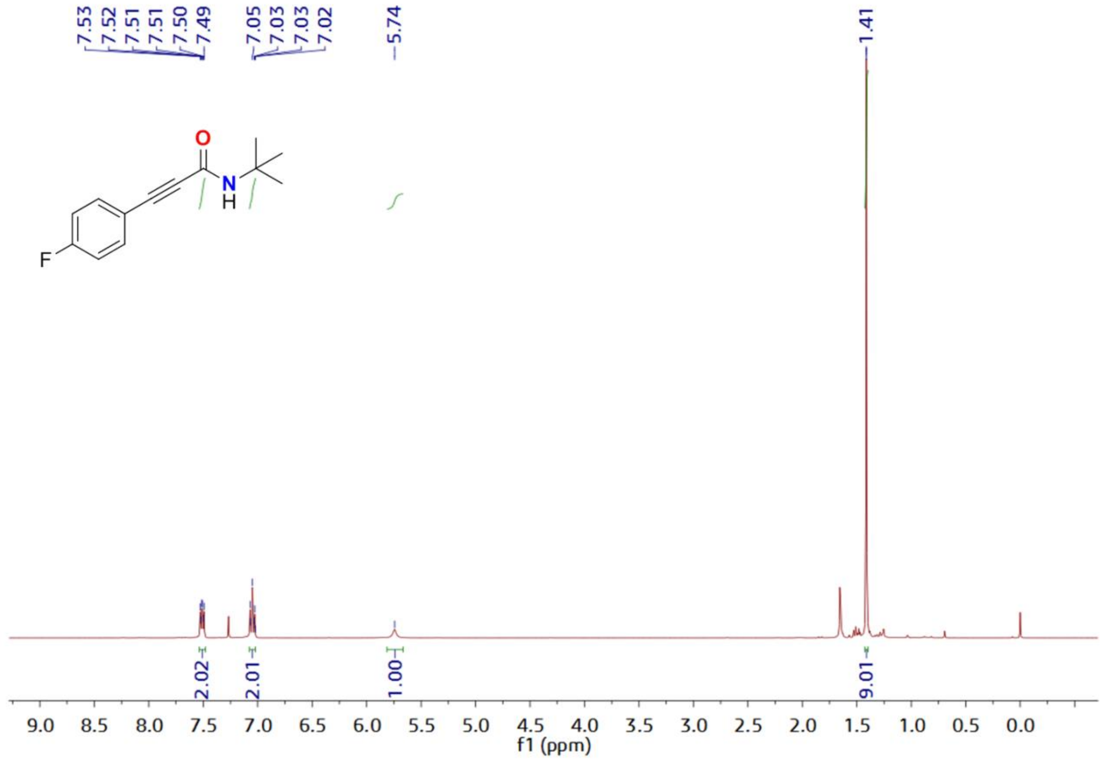

^{13}C NMR of compound 3f

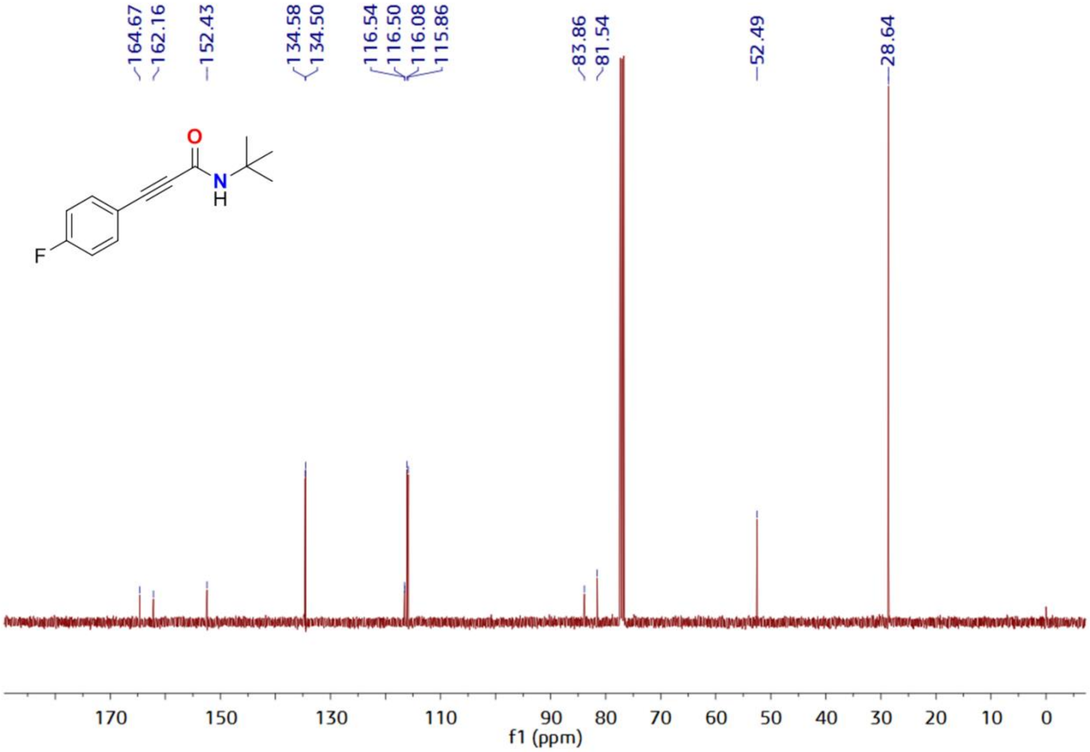

^{1}H NMR of compound 3g

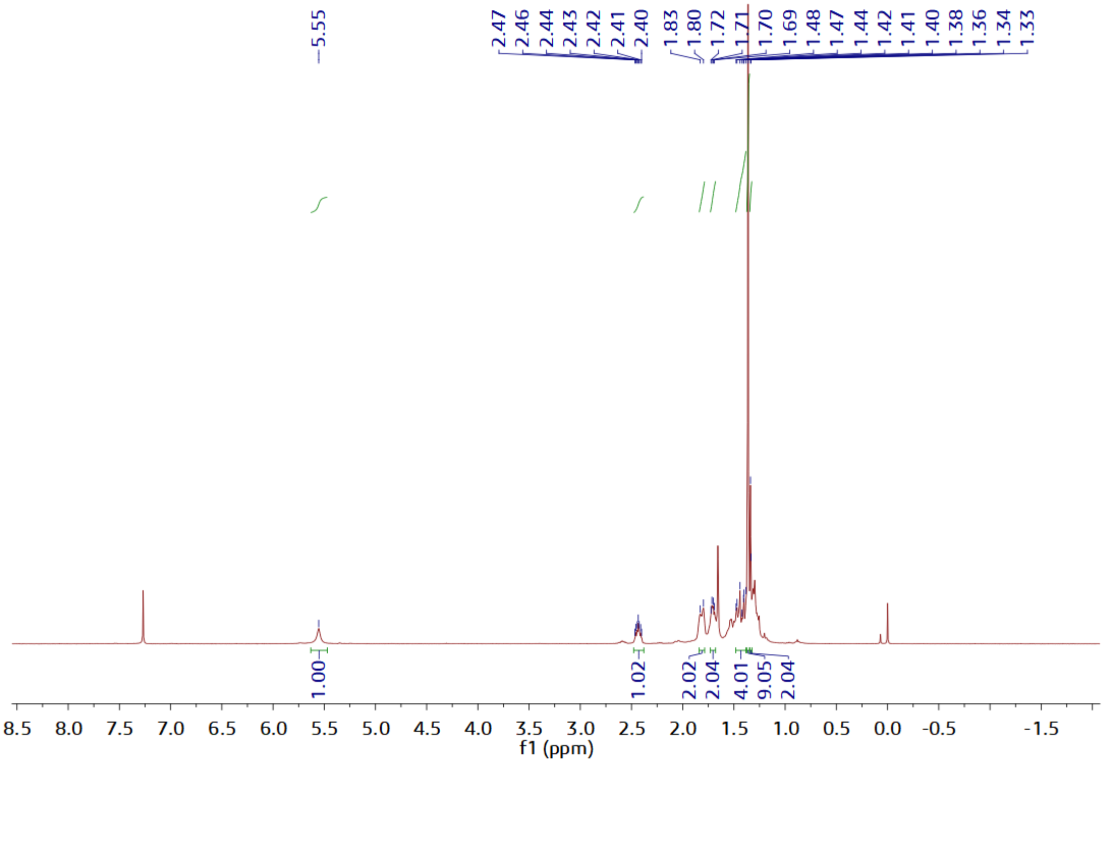

^{13}C NMR of compound 3g

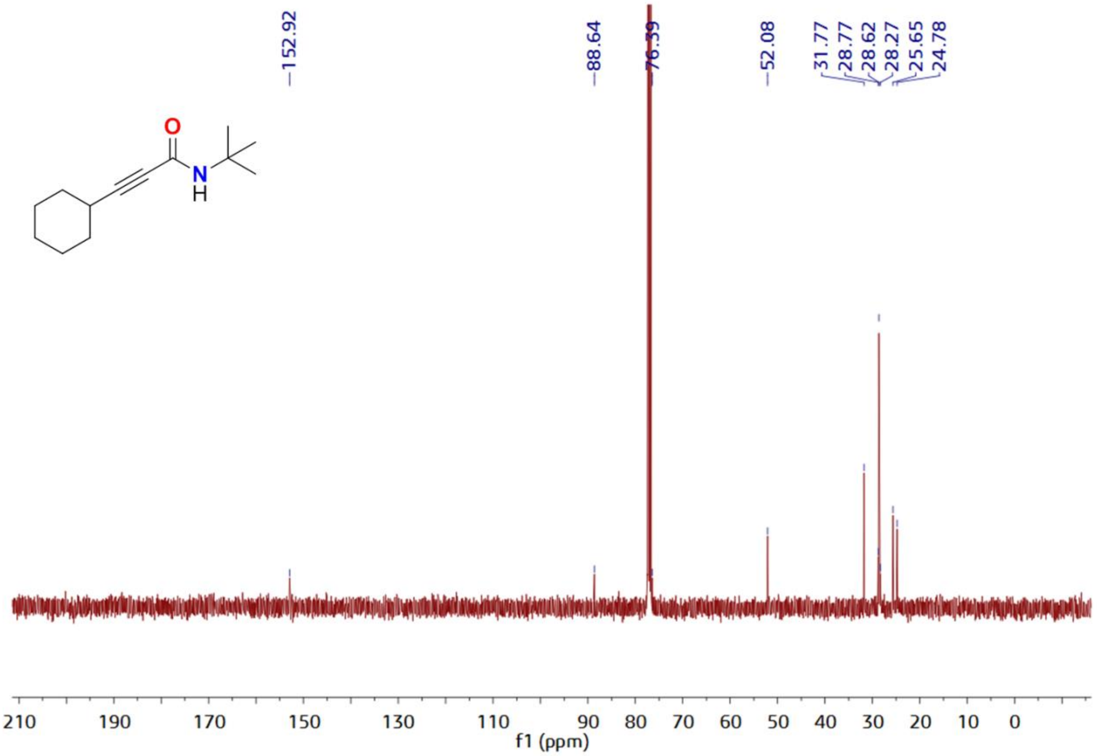

^{1}H NMR of compound 3h

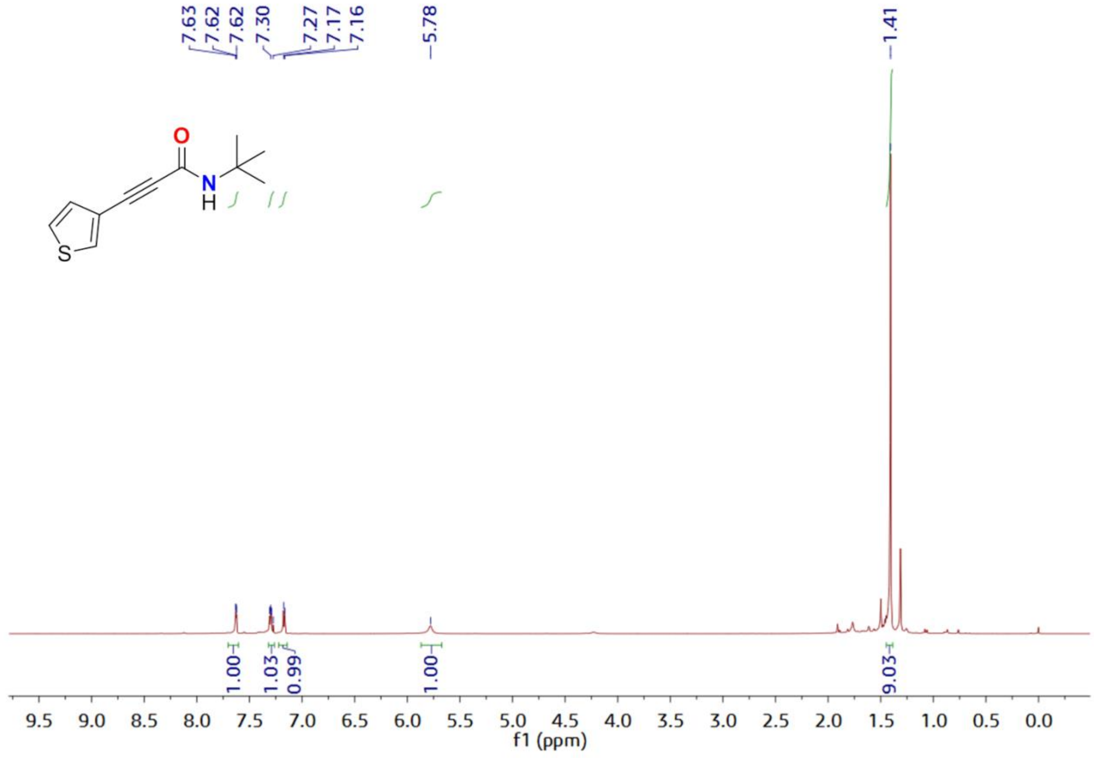

^{13}C NMR of compound 3h

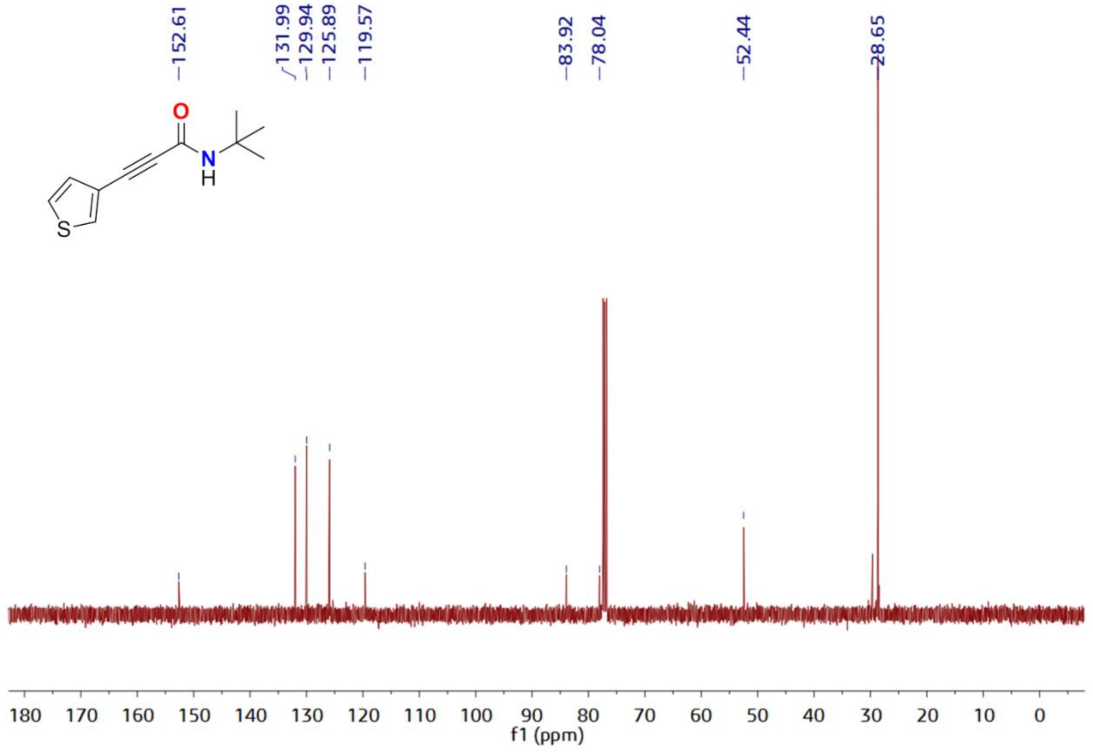

^{1}H NMR of compound 3i

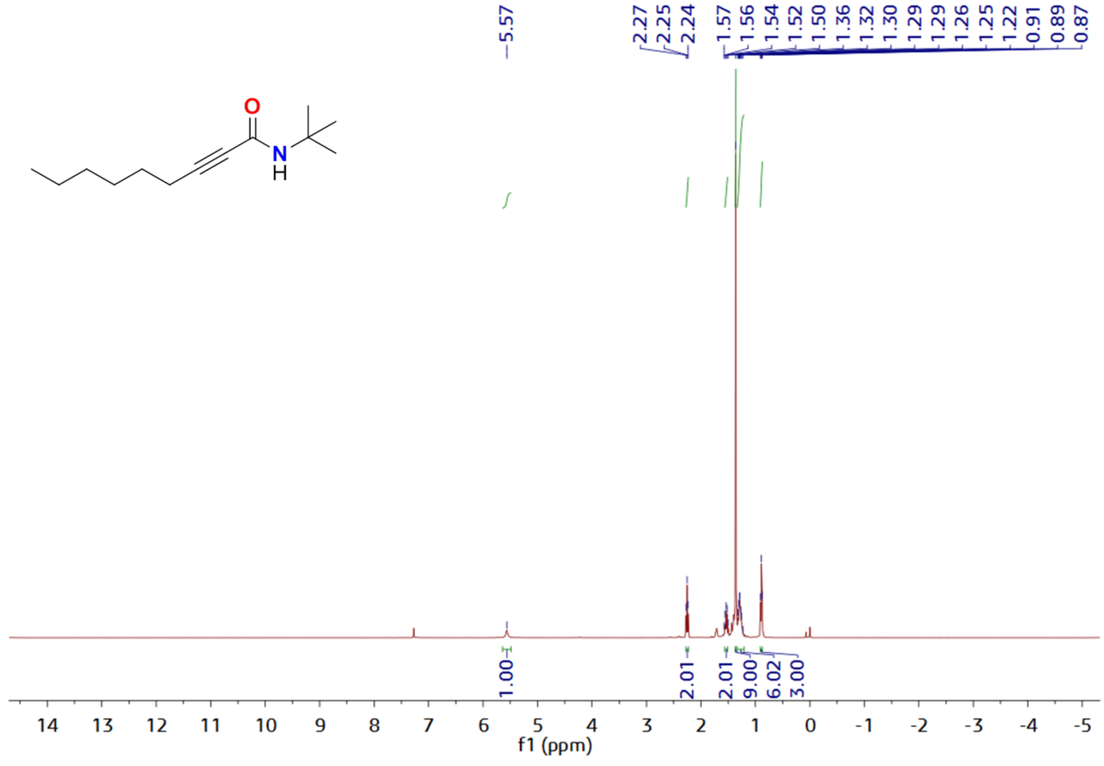

^{13}C NMR of compound 3i

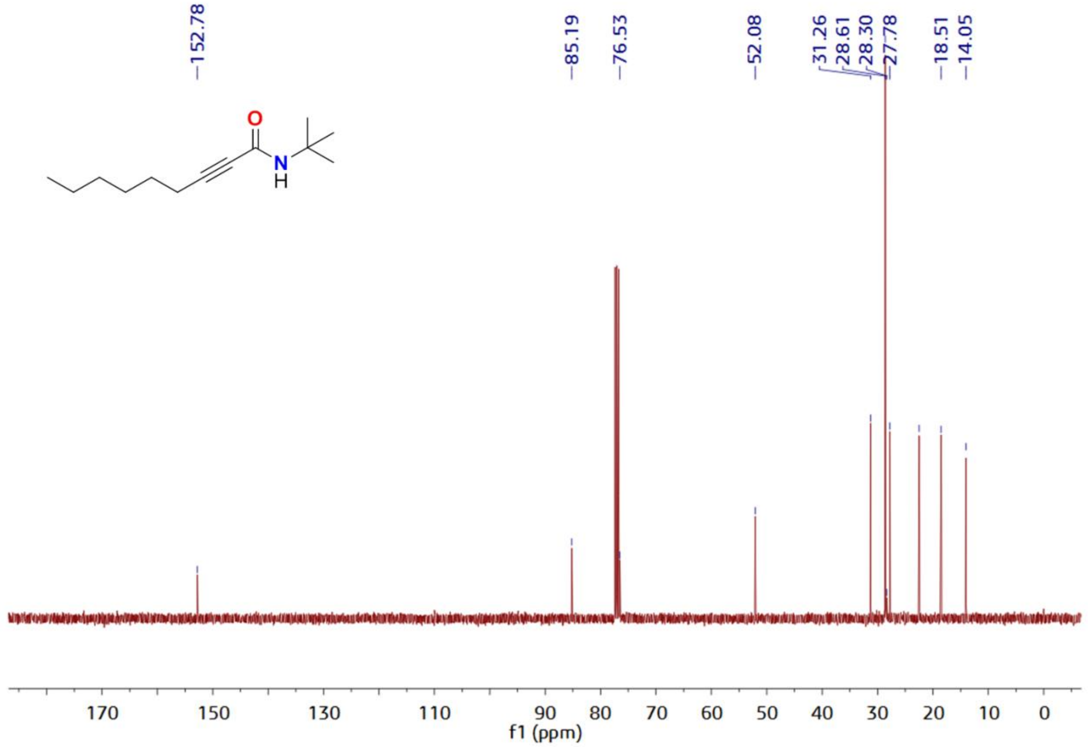

^{1}H NMR of compound 3j

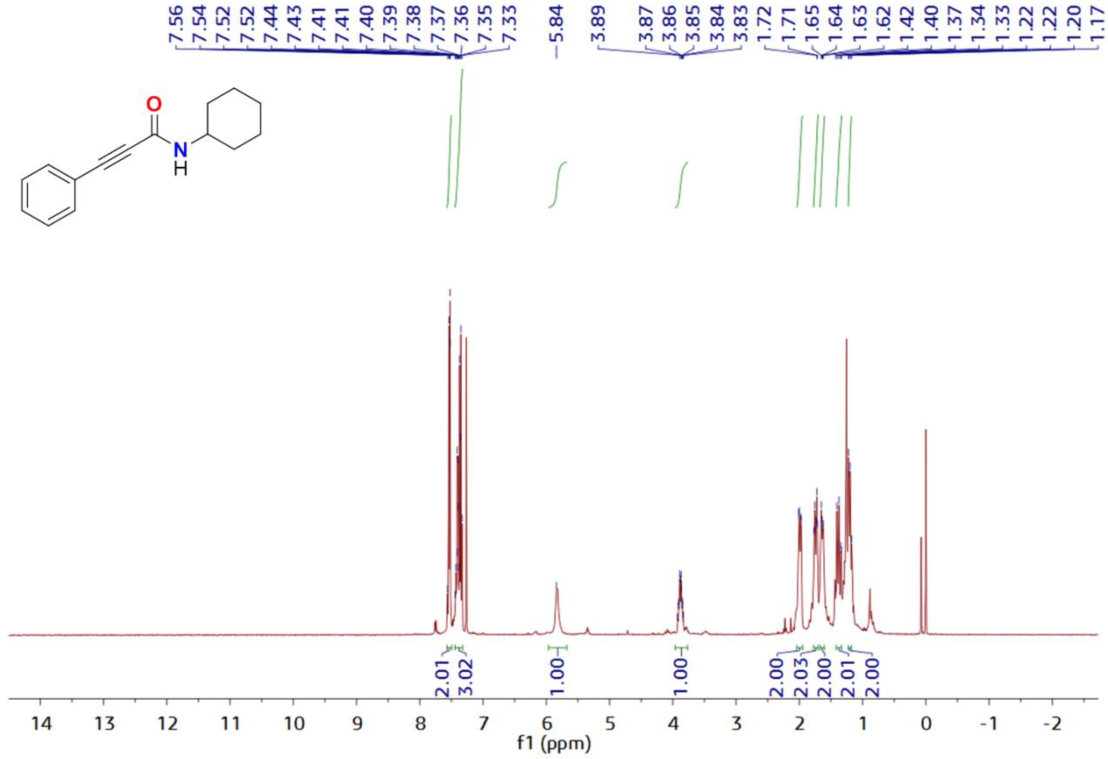

^{13}C NMR of compound 3j

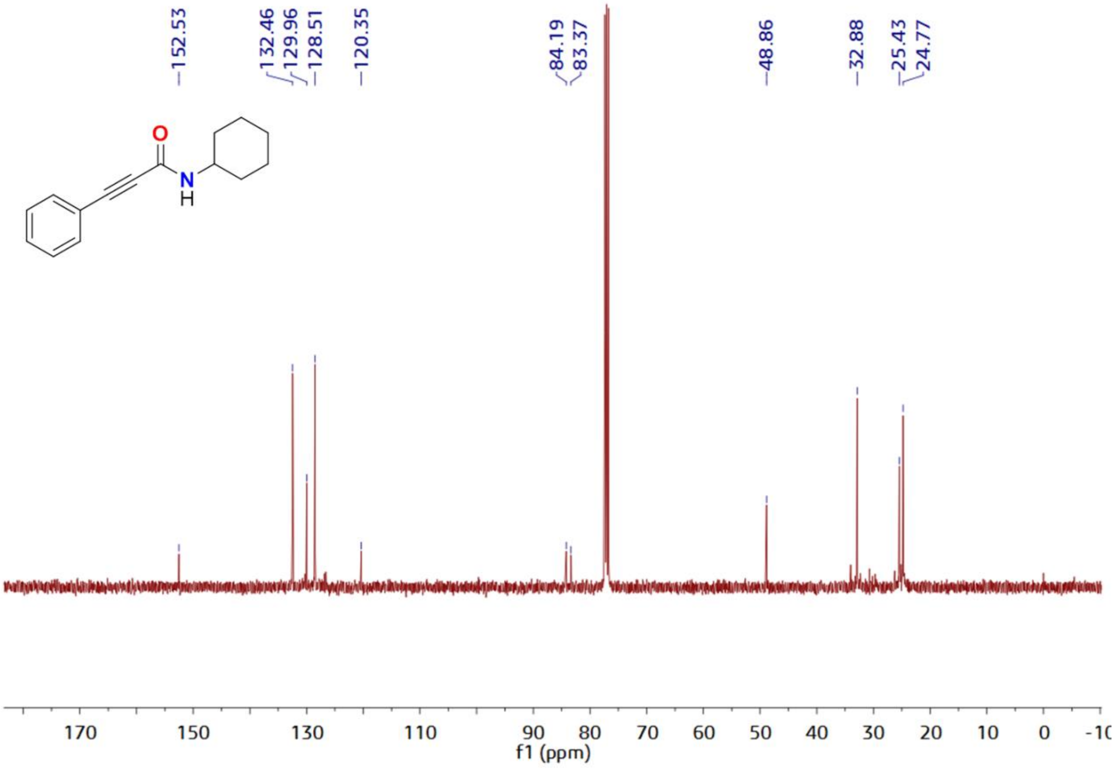

^{1}H NMR of compound 3k

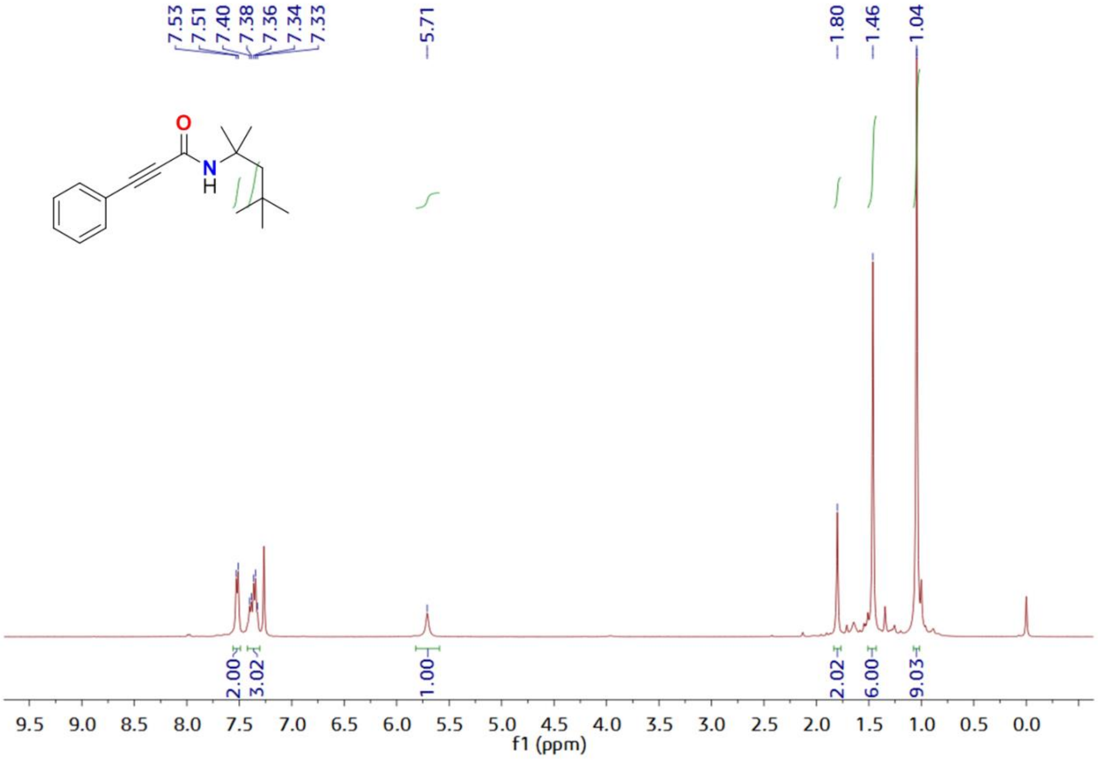

^{13}C NMR of compound 3k

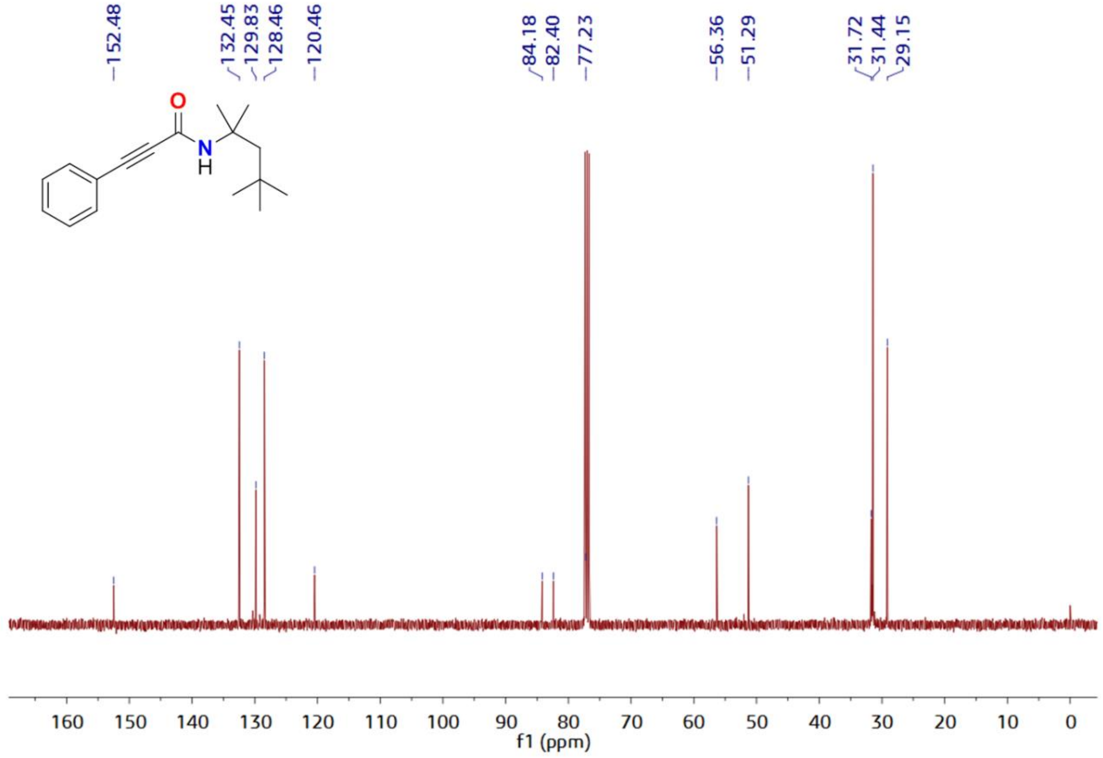

References

[1] H. Hu, B. Guan, B. Xia, X. W. Lou, *J. Am. Chem. Soc.* **2015**, *137*, 5590-5595.

[2] M. J. Frisch, G. W. Trucks, H. B. Schlegel, G. E. Scuseria, M. A. Robb, J. R. Cheeseman, G. Scalmani, V. Barone, G. A. Petersson, H. Nakatsuji, X. Li, M. Caricato, A. V. Marenich, J. Bloino, B. G. Janesko, R. Gomperts, B. Mennucci, H. P. Hratchian, J. V. Ortiz, A. F. Izmaylov, J. L. Sonnenberg, D. Williams-Young, F. Ding, F. Lipparini, F. Egidi, J. Goings, B. Peng, A. Petrone, T. Henderson, D. Ranasinghe, V. G. Zakrzewski, J. Gao, N. Rega, G. Zheng, W. Liang, M. Hada, M. Ehara,K. Toyota, R. Fukuda, J. Hasegawa, M. Ishida, T. Nakajima, Y. Honda, O.Kitao, H. Nakai, T. Vreven,K. Throssell, J. A. Montgomery, Jr., J. E. Peralta, F. Ogliaro, M. J. Bearpark, J. J. Heyd, E. N. Brothers,K. N.Kudin, V. N. Staroverov, T. A.Keith, R.Kobayashi, J. Normand,K. Raghavachari, A. P. Rendell, J. C. Burant, S. S. Iyengar, J. Tomasi, M. Cossi, J. M. Millam, M.Klene, C. Adamo, R. Cammi, J. W. Ochterski, R. L. Martin,K. Morokuma, O. Farkas, J. B. Foresman, D. J. Fox, *Gaussian Inc Wallingford CT* **2013**.

[3] C. Lee, W. Yang, R. G. Parr, *Phys. Rev., B Condens. Matter* **1988**, *37*, 785-789.

[4] A. D. Becke, *J. Chem.Phys.* **1993**, *98*, 5648-5652.

[5] M. Dolg, U. Wedig, H. Stoll, H. Preuss, *J. Chem. Phys.* **1987**, 86, 866-872.

[6] M. Dolg, H. Stoll, H. Preuss, *J. Chem. Phys.* **1989**, *90*, 1730-1734.

[7] A. V. Marenich, C. J. Cramer, D. G. Truhlar, *J. Phys. Chem. B* **2009**, *113*, 6378-6396.

[8] Y. Zhao, D. G. Truhlar, *J. Chem.Phys.* **2006**, *125*, 194101.

[9] Y. Dang, S. Qu, Y. Tao, X. Deng, Z. X. Wang, *J. Am. Chem. Soc.* **2015**, *137*, 6279-6291.

[10] X. Hong, Y. Liang, K. N. Houk, *J. Am. Chem. Soc.* **2014**, *136*, 2017-2025.

[11] A. Nagarsenkar, S. K. Prajapti, S. D. Guggilapu, B. Nagendra Babu, *Org. Lett.* **2015**, *17*, 4592-4595.

[12] G. K. Rathod, R. Jain, *J. Org. Chem.* **2023**, *88*, 7219-7227.

[13] L. Zeng, H. Li, J. Hu, D. Zhang, J. Hu, P. Peng, S. Wang, R. Shi, J. Peng, C.-W. Pao, J.-L. Chen, J.-F. Lee, H. Zhang, Y.-H. Chen, A. Lei, *Nat. Catal.* **2020**, *3*, 438-445.

[14] N. L. Hughes, C. L. Brown, A. A. Irwin, Q. Cao, M. J. Muldoon, *ChemSusChem* **2017**, *10*, 675-680.

[15] R. Suleiman, J. Tijani, B. E. Ali, *Appl. Organometal. Chem.* **2010**, *24*, 38-46.
[16] S. T. Gadge, M. V. Khedkar, S. R. Lanke, B. M. Bhanage, *Adv. Synth. Catal.* **2012**, *354*, 2049-2056.
[17] H. Yin, T. Skrydstrup, *J. Org. Chem.* **2017**, *82*, 6474-6481
[18] Z. Huang, Y. Dong, Y. Li, M. Makha, Y. Li, *ChemCatChem* **2019**, *11*, 5236-5240
[19] Y. Dong, S. Sun, F. Yang, Y. Zhu, W. Zhu, H. Qiao, Y. Wu, Y. Wu, *Org. Chem. Front.* **2016**, *3*, 720-724
[20] J. C. Arango-Daza, J. R. Cabrero-Antonino, R. Adam, ChemSusChem **2024**, *17*, e202400331
[21] X. Zhao, X. Feng, F. Chen, S. Zhu, F.-L. Qing, L. Chu, *Angew. Chem. Int. Ed.* **2021**, *60*, 26511-26517
[22] G. K. Rathod, R. Jain, *J. Org. Chem.* **2023**, *88*, 7219-7227.
[23] D. A. Petrone, I. Franzoni, J. Ye, J. F. Rodríguez, A. I. Poblador-Bahamonde, M. LautensPetrone, *J. Am. Chem. Soc.* **2017**, *139*, 3546-3557.
